# Notoginsenoside R1 mitigates UVB-induced skin sunburn injury through modulation of *N*^4^-acetylcytidine and autophagy

**DOI:** 10.1186/s13020-025-01270-3

**Published:** 2025-12-18

**Authors:** Shuyun Liang, Xiaokang Liu, Yuting Yang, Fangyuan Zhang, Xiaobo Sun, Tong Zhang, Dean Guo, Jiyu Gong, Zizhao Yang

**Affiliations:** 1 School of Pharmaceutical Sciences, Changchun University of Chinese Medicine, Changchun, 130117 China; 2 School of Pharmacy, Shanghai University of Traditional Chinese Medicine, Shanghai, 201203 China; 3 Zhongshan Institute for Drug Discovery, Shanghai Institute of Materia Medica, Chinese Academy of Sciences, Zhongshan, 528400 China; 4 Institute of Medicinal Plant Development, Peking Union Medical College & Chinese Academy of Medical Sciences, Beijing, 100193 China; 5 Center for Laboratory Animal Service & Experiments, The Seventh People’s Hospital of Shanghai University of Traditional Chinese Medicine, Shanghai, 200137 China; 6 Shanghai International Standardization Research Institute of Traditional Chinese Medicine, Shanghai University of Traditional Chinese Medicine, Shanghai, 201203 China

**Keywords:** Panax Notoginseng Saponins (PNS), Notoginsenoside R1 (NGR1), *N*-acetyltransferase 10 (NAT10), Ultraviolet B (UVB) Radiation, PI3K/AKT/mTOR Signaling Pathway, Autophagic Process

## Abstract

**Background:**

In recent years, skin sunburn injury caused by UVB has become a growing concern. Although PNS have demonstrated potential in alleviating this condition, the precise mechanisms involved remain incompletely elucidated.

**Purpose:**

This study was designed with three primary objectives. First, to apply network pharmacology-based predictive approaches to elucidate the mechanisms underlying PNS-mediated protection against UVB-induced skin sunburn injury. Second, to systematically analyze the chemical profile of PNS through UHPLC-Q-Orbitrap-MS/MS. Third, to conduct a comprehensive assessment of the pharmacodynamic properties of NGR1, a major bioactive constituent of PNS.

**Methods:**

The chemical constituents of PNS were analyzed qualitatively and quantitatively using UHPLC and UHPLC-Q-Trap-MS/MS. Network pharmacology approaches were employed to identify the core molecular targets and potential mechanisms through which PNS alleviates UVB-induced sunburn injury. To evaluate the therapeutic effects of PNS and NGR1, an in vivo model was established using nude mice, while mechanistic studies were conducted in HaCaT cells to elucidate the underlying signaling pathways.

**Results:**

A total of 16 primary saponins in PNS were successfully identified and quantified. Through network pharmacology analysis, 49 crucial molecular targets associated with PNS in the context of UVB-induced skin sunburn injury were revealed. Treatment with PNS and NGR1 ameliorated signs of photoaging via multiple mechanisms, including suppression of inflammatory responses, boosting antioxidant capacity, inhibition of the PI3K/AKT/mTOR signaling cascade, and regulation of proteins involved in maintaining cellular homeostasis. In HaCaT cells, PNS and NGR1 exert protective effects against apoptosis by modulating proteins associated with cellular homeostasis and autophagy. Both compounds counteracted the UVB-induced reduction in NAT10 expression. The degradation of NAT10, potentially mediated by the autophagy pathway involving key selective adaptors such as NBR1 and p62, may occur under both basal and UVB-exposed conditions.

**Conclusion:**

PNS and NGR1 demonstrate promising therapeutic potential for the treatment of UVB-induced skin sunburn injury. Their capacity to mitigate photodamage via multiple mechanisms, such as inhibition of key signaling pathways, regulation of apoptosis and autophagy, and modulation of NAT10 expression, lays a strong foundation for future clinical studies on topical applications of PNS and NGR1, while also providing valuable insights into their preventive and curative effects.

**Graphical Abstract:**

PNS prevents cell apoptosis and autophagy by suppressing the activation of the PI3K/AKT/mTOR signaling pathway, whereas NGR1 enhances the activity of RNA acetyltransferase NAT10 to exert its protective effects. Their combined action contributes to the recovery from UVB-induced skin sunburn injury.

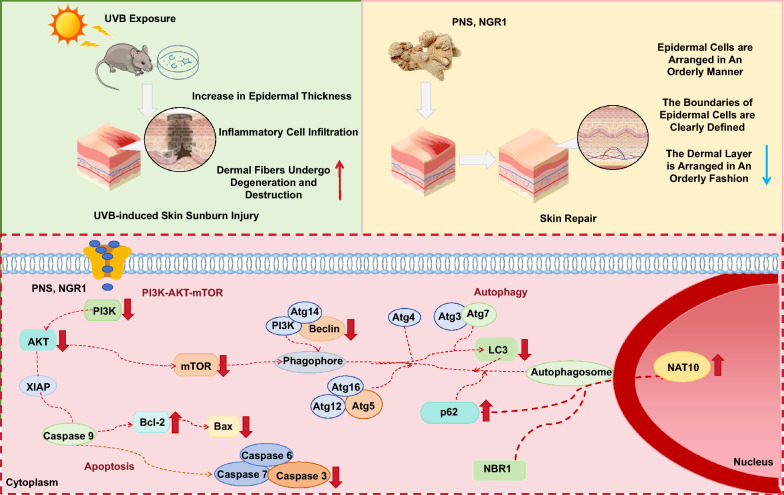

**Supplementary Information:**

The online version contains supplementary material available at 10.1186/s13020-025-01270-3.

## Highlights

1. In a nude mouse model, the synergistic effect of PNS and NGR1 significantly alleviates skin injury caused by UVB-induced sunburn.
2. Upon UVB exposure, PNS effectively suppresses the activation of the PI3K/AKT/mTOR signaling cascade.
3. In response to UVB radiation, PNS modulates the expression of key proteins involved in apoptosis and autophagy.
4. Under UVB stress, NGR1 promotes the autophagic clearance of NAT10 via the recruitment of selective adaptors NBR1 and p62.

**Supplementary Information:**

The online version contains supplementary material available at 10.1186/s13020-025-01270-3.

## Introduction

Ultraviolet B (UVB) radiation, known as the most biologically potent fraction of sunlight, possesses high energy and notable skin-penetrating ability. Upon exposure, UVB triggers a range of cutaneous injuries and initiates a complex cascade of biological responses, leading to extensive changes in both the structure and function of the skin. The resulting damage, commonly termed sunburn, constitutes the initial and mildest form of photodamage. Without appropriate intervention, such damage may evolve into xeroderma pigmentosum (XP) [1], a genetic disorder associated with a markedly elevated risk of squamous cell carcinoma (SCC), particularly due to impairments in the global genome nuclear excision repair (GGR) mechanism [2, 3]. Prolonged or repeated UVB exposure not only causes direct harm to skin cells but also promotes premature skin injuries and significantly heightens susceptibility to skin malignancies.

Skin sunburn injury results from a combination of intrinsic and extrinsic factors, manifesting in visible alterations including skin roughness, uneven pigmentation, and loss of elasticity. While intrinsic aging occurs naturally with time, extrinsic aging is largely accelerated by environmental stressors, particularly UV radiation. Exposure to UVB rays induces epidermal hyperplasia and compromises skin elasticity due to chronic sunlight exposure [4, 5]. This form of photodamage triggers cellular stress and inflammation, frequently contributing to the emergence of wrinkles, dyspigmentation, and laxity [6]. Prolonged or intense UVB irradiation can promote skin lesion formation, hasten the photoaging process, and elevate the risk of tumor development [7]. Consequently, there is growing global interest in discovering and developing effective therapeutic agents that can repair and protect against UV-induced skin sunburn injury.

In recent years, an increasing body of research has underscored the positive impact of natural therapeutics on skin health, fueling a rising interest in developing plant-derived agents for the prevention and treatment of UVB-induced cutaneous damage. Natural products contain various bioactive constituents—such as flavonoids, isoflavones, and ginsenosides—that have demonstrated protective effects against photodamage [8–12]. Among these, saponins are widely acknowledged as safe and potent natural compounds with anti-aging properties in dermatological use, with extensive evidence supporting their role in skin protection and regeneration. For instance, ginsenoside Rg1 has been found to counteract UVB-triggered glucocorticoid resistance in keratinocytes by activating the Nrf2/HDAC2 signaling axis [13]. Similarly, ginsenoside Rc treatment effectively reduces reactive oxygen species (ROS) generation and inhibits the UVB-induced elevation of pro-MMP-2 and pro-MMP-9 in irradiated keratinocytes [14–17]. Additionally, our findings reveal a strong link between UVB exposure in HaCaT cells and the onset of apoptosis [18, 19]. UVB-mediated cellular injury predominantly involves apoptotic pathways, where radiation induces ROS accumulation and DNA lesions, thereby initiating the mitochondrial-dependent apoptosis cascade. Moreover, the phosphorylation of RIPK3 kinase acts as an amplifier and regulatory node in this process, influencing the magnitude of cell death [20].

*Panax Notoginseng Saponins* (PNS), an extract derived from Panax Notoginseng (Burk.) F.H. Chen of the Araliaceae family is a complex bioactive compound that has attracted considerable attention in medical research. It possesses a wide range of pharmacological properties, including enhancement of hematopoietic function, modulation of immune responses, reduction of inflammation, and inhibition of aging processes, making it a key focus of clinical investigations. Officially listed in the 2020 edition of the Chinese Pharmacopoeia, PNS has demonstrated notable clinical efficacy in the treatment of traumatic injuries. In the context of skin damage, PNS exhibits therapeutic potential, particularly in alleviating UVB-induced injury. However, further comprehensive scientific investigation is required to fully elucidate its underlying cellular repair mechanisms. Notoginsenoside R1 (NGR1), a unique constituent of total saponins derived from Panax Notoginseng, exhibits diverse pharmacological activities. Accumulating evidence has demonstrated its therapeutic potential in atopic dermatitis, as well as its ability to promote angiogenesis and accelerate wound healing through activation of the Notch signaling pathway [21]. NGR1 demonstrates therapeutic efficacy in promoting skin wound healing [22]. Compared to PNS, NGR1, as a single active compound, exhibits more defined pharmacological effects, enabling a clearer structure–activity relationship. Ginsenoside C-Mx, isolated from the total saponins of Panax Notoginseng leaves, has been shown to suppress UVB-induced expression of intracellular reactive oxygen species (ROS), MMP-1, and interleukin-6 (IL-6). It effectively counteracts UVB-induced degradation of type I procollagen by modulating the TGF-β/Smad signaling pathway [23]. Saponin components derived from Panax Notoginseng have been widely used in the treatment of skin injuries. Although PNS is recognized for its protective effects against UVB-induced sunburn, its underlying repair mechanisms remain to be fully elucidated.

RNA modifications exert significant effects on molecular functions, and accumulating evidence underscores the therapeutic potential of targeting these pathways in cancer [24]. The *N*^4^-acetylcytidine (ac^4^C) modification, a prevalent form of mRNA chemical modification, plays a crucial role in regulating mRNA stability and translational efficiency. However, the specific implications of ac^4^C modification in disease pathogenesis remain incompletely understood [25]. UVB exposure in skin cells can lead to DNA damage, and RNA acetylation plays a role in regulating cellular repair capacity by modulating the expression of DNA damage repair-related genes. NAT10 is the enzyme responsible for ac^4^C RNA modification and plays a key role in RNA regulatory networks [26]. This study elucidates the mechanism by which NAT10-mediated mRNA ac^4^C modification regulates UVB-induced skin sunburn injury.

Network pharmacology, an interdisciplinary field that integrates principles from systems biology and network informatics, has gained considerable attention in recent years for its application in developing novel therapeutics, particularly in uncovering synergistic interactions among multiple components, pathways, and targets [27–29]. The integration of large-scale datasets facilitates the identification of novel drug targets and elucidation of underlying molecular mechanisms [30–32]. Currently, network pharmacology is increasingly utilized to explore the therapeutic potential of traditional Chinese medicine across a wide range of diseases.

This study was designed to evaluate the therapeutic efficacy of PNS and NGR1 in alleviating UVB-induced skin sunburn injury and to elucidate the underlying molecular mechanisms. First, Ultra-High-Performance Liquid Chromatography coupled with Quadrupole Orbitrap Tandem Mass Spectrometry (UHPLC-Q-Orbitrap-MS/MS) was employed to analyze the chemical composition of PNS. Subsequently, a network pharmacology approach was used to predict potential therapeutic targets and associate molecular mechanisms of PNS in mitigating such damage, followed by experimental validation. The objective of this research is to explore the mechanistic basis of PNS and NGR1 in treating UVB-induced skin sunburn injury and to provide supporting evidence for the development of related therapeutic or cosmetic products.

## Materials and methods

### Chemical reagent

The preparation method for PNS is detailed in the corresponding monograph of the 2020 edition of the Chinese Pharmacopoeia. Briefly, Panax Notoginseng is ground into a coarse powder and extracted with 70% ethanol. The resulting mixture is filtered, and the filtrate is concentrated under reduced pressure. The concentrate is then loaded onto a column packed with non-polar or weakly polar styrene-based macroporous adsorption resin. After washing with water and discarding the aqueous fraction, the resin is eluted with 80% ethanol. The elevator is subsequently concentrated under vacuum, decolorized, further concentrated, and purified to obtain the final extract, which is then dried to yield the PNS product. NGR1, a distinctive bioactive constituent of Panax Notoginseng, is one of the key components isolated from the total saponin fraction and plays a significant role within this complex mixture. PNS with a purity exceeding 75% was acquired from the China Institute for Food and Drug Control (batch number: 110870-202105). Reference standards of NGR1 (C_47_H_80_O_18_, batch: 110745-202322), Ginsenoside Rg1 (C_42_H_72_O_14_, batch: 110703-202436), Rb1 (C_54_H_92_O_23_, batch: 110704-202331), Rd (C_48_H_82_O_18_, batch: 111818-202305), and Re (C_48_H_82_O_18_, batch: 110754-202330), each with purity >95%, were also obtained from the same institution. All-Trans Retinoic Acid (ATRA) was obtained from Sigma-Aldrich (St. Louis, MO, USA). Formic acid for mass spectrometry analysis was supplied by Sigma-Aldrich (St. Louis, MO, USA), and HPLC-grade acetonitrile and methanol were purchased from Fisher Scientific (Pittsburgh, PA, USA).

### UHPLC-Q-Orbitrap-MS/MS

Preparation of PNS Sample Solution: Accurately weigh 10 mg of PNS and dissolve it in 10 mL of 70% (*v*/*v*) methanol–water solution. Subject the mixture to ultrasonic extraction for 15 min under ambient conditions. Afterwards, pass the extract through a 0.22 μm membrane filter. Collect the filtrate and transfer it into a vial for subsequent analysis by UHPLC-Q-Orbitrap-MS/MS.

Chromatographic separation was carried out using an Ultimate 3000 UHPLC system (Thermo, San Jose, CA, USA) equipped with a Supelco C18 column (3.0 × 50 mm, 2.7 μm; Sigma-Aldrich). The column temperature kept constant at 35 °C during the entire run. The mobile phase consisted of acetonitrile (A) and water (B). A gradient elution program was applied as follows: initial isocratic elution with 80% B from 0 to 20 min, followed by a linear gradient down to 54% B from 20 to 45 min, then further decreased to 45% B between 45 and 55 min, held at 45% B from 55 to 60 min, subsequently increased stepwise to 80% B from 60 to 65 min, and finally re-equilibrated at 80% B for 5 min. The flow rate was maintained at 0.4 mL/min, and an injection volume of 5 μL was used for each sample.

Mass spectrometry was conducted using a Q-Orbitrap-MS/MS system (Thermo, San Jose, CA, USA) equipped with an electrospray ionization (ESI) source operating in negative ion mode. The ion source was operated with the following parameters: sheath gas flow at 40 (arbitrary units), auxiliary gas flow at 10 (arbitrary units), and sweep gas flow at 1 (arbitrary unit). The S-Lens RF level was set to 55%, while the capillary voltage was maintained at −3.5 kV and the capillary temperature held constant at 350 °C. Full-scan MS spectra were collected in centroid mode over an *m*/*z* range of 150–1500 with a resolution of 70,000. The automatic gain control (AGC) target was configured to 1 × 10^6^, and the maximum ion injection time was limited to 100 ms. For tandem mass spectrometry, data were acquired in Full-MS/data-dependent MS^2^ (ddMS^2^) mode using the following settings: resolution of 17,500, AGC target of 1 × 10^5^, maximum injection time of 50 ms, loop count of 5, Top N set to select the five most intense precursor ions, an isolation window of 4.0 m/*z*, and stepped collision energies of 35, 45, and 55 eV applied for fragmentation.

### UHPLC-Q-Trap-MS/MS

Preparation of PNS Sample Solution: weigh 1 mg of PNS and dissolve it in 10 mL of 70% (*v*/*v*) methanol–water solution at room temperature. Perform ultrasonic extraction for 15 min, followed by filtration through a 0.22 μm membrane filter to obtain a clear sample solution. For the standard solution preparation, accurately weigh appropriate quantities of reference compounds NGR1, Ginsenoside Rg1, Ginsenoside Rb1, Ginsenoside Re, and Ginsenoside Rd. Dissolve each in 1 mL of 70% (*v*/*v*) methanol–water and subject the mixture to ultrasonic extraction for 15 min under ambient conditions to yield a mixed standard solution with a final concentration of 100 ng/mL. This solution is then filtered through a 0.22 μm membrane filter. Both the sample and standard filtrates are transferred into designated vials for subsequent analysis using UHPLC-Q-Trap-MS/MS.

Chromatographic separation was performed in reversed-phase mode using an ultra-high-performance liquid chromatography (UPLC) system interfaced with a quadrupole mass spectrometer (QTRAP 6500; ABSciex, Framingham, MA). The analytical separation was achieved on a Waters ACQUITY BEH C18 column (100 mm × 2.1 mm, 1.7 μm; Waters Corporation, Milford, MA, USA). The mobile phase comprised 0.1 mmol/L ammonium acetate in water (solvent A) and acetonitrile (solvent B), eluted at a flow rate of 0.35 mL/min. The column was kept at a constant temperature of 35 °C during the entire run.

Quantitative analysis of multiple components in PNS was performed using a gradient elution program with the following profile: mobile phase B was maintained at 30% from 0 to 2.0 min, then gradually increased to 37% by 5.0 min, further elevated to 42% at 9.0 min, and raised to 47% at 14.0 min. By 17.0 min, the concentration of mobile phase B reached 51%, followed by a rapid increase to 95% between 20.0 and 21.0 min. Finally, the system was re-equilibrated by returning to the initial condition of 30% B from 22.0 to 23.0 min.

The mass spectrometer was operated in negative ion mode with optimized settings: an ion spray voltage of −4.5 kV, ion source temperature maintained at 550 °C, nebulizer gas (Gas 1) pressure at 20 psi, heater gas (Gas 2) pressure at 30 psi, and curtain gas pressure kept at 10 psi. To ensure precise quantification of the PNS components, multiple reaction monitoring (MRM) was employed as the detection method throughout the analytical run.

### HPLC analysis

Preparation of PNS Sample Solution: Precisely weigh 3 mg of PNS at ambient temperature. Perform ultrasonic extraction using 1 mL of a 70% (*v*/*v*) methanol–water mixture for 15 min. After sonication, filter the extract through a 0.22 μm membrane filter to obtain a clear sample solution suitable for HPLC analysis.

Preparation of Standard Solution: Accurately weigh fifteen saponins—NGR1, Ginsenoside Rg1, Ginsenoside Rb1, Ginsenoside Re, Ginsenoside Rd, Notoginsenoside C, Pseudoginsenoside RT5, Ginsenoside Rf, Momordicoside A, Ginsenoside F2, Ginsenoside Rb2, Ginsenoside Rg3, Gypenoside XVII, Notoginsenoside Fe, and Ginsenoside Rh4. Under the same room temperature conditions, dissolve each in 1 mL of 70% (*v*/*v*) methanol–water solution and subject to 15 min of ultrasonic treatment. Combine and prepare into a mixed standard solution with a final concentration of 800 mg/mL. This solution is also filtered through a 0.22 μm membrane prior to being transferred into a dedicated vial for HPLC analysis.

Chromatographic Conditions: The mobile phase system consists of water as phase A and acetonitrile as phase C, delivered at a flow rate of 0.35 mL/min with the column temperature held constant at 35 °C. For multi-analyte quantification of PNS components, the following gradient program is applied: 0–3.0 min, 81% C; 3.0–9.0 min, 79% C; 16.0 min, 74% C; 30.0 min, 72% C; 33.0 min, 65.0% C; 38.0 min, 62.0% C; 45.0 min, 59.0% C; 50.0 min, transition to 55% B (implied from context); 57.0 min, reduce to 5.0% C; and finally, re-equilibrate at 81.0% C by 62.0 min to restore initial conditions.

### Cell culture

The human keratinocyte cell line HaCaT (Catalog No.: ATCC CRL-2404) and the human embryonic kidney cell line 293 T (Catalog No.: ATCC CRL-3216) were obtained from Proteintech (Wuhan, China). Cells were maintained in Dulbecco’s Modified Eagle Medium (DMEM, Gibico, USA) enriched with 10% fetal bovine serum (FBS, Gibico, USA), along with 100 units/mL penicillin and 100 μg/mL streptomycin (both from Gibico, USA). Cultures were incubated under standard conditions.

### Animal experiments

Six-week-old female BALB/c nude mice were obtained from Beijing Vital River Laboratory Animal Technology Co., Ltd. (China). After arrival, the animals were housed under specific pathogen-free (SPF) conditions for a 1-week acclimatization period, with environmental temperature kept at 15–25 °C and relative humidity maintained between 40 and 65%. A 12-h light/dark cycle was strictly controlled, and all animals had free access to standard rodent diet and fresh water throughout the experimental period. A total of forty mice were randomly assigned to five experimental groups: the untreated control group (sham), the UVB-induced model group treated with blank cream base (UVB + vehicle), the positive control group receiving 5 mM ATRA cream combined with UVB exposure (UVB + 5 mM ATRA), a low-dose PNS treatment group administered with 2.5 mM PNS cream and UVB irradiation (UVB + PNS-Low), and a high-dose PNS group treated with 5.5 mM PNS cream under UVB exposure (UVB + PNS-High) [33]. Additionally, two subgroups were included for NGR1 evaluation: the low-dose NGR1 group (UVB + NGR1-Low) treated with 2.5 mM NGR1 cream and the high-dose NGR1 group (UVB + NGR1-High) treated with 5.5 mM NGR1 cream, both subjected to UVB radiation [34]. Treatments were applied topically. UVB irradiation was delivered at 80 mJ/cm^2^, 5–6 times per week for 1 week. After the final dose, mice were fasted for 12 h, anesthetized, and euthanized. Blood samples and dorsal skin tissues were harvested for subsequent biochemical and histological analyses. All animal procedures were conducted in compliance with the guidelines approved by the Institutional Animal Care and Use Committee (IACUC) of Changchun University of Chinese Medicine, under protocol number 2024299, granted on June 13, 2024.

### Hematoxylin and eosin (H&E) staining

Skin specimens were harvested from each group of nude mice and processed for histopathological analysis according to standard procedures. The collected tissues were fixed in 4% neutral buffered formalin for 24 h, then dehydrated under vacuum and embedded in paraffin wax. Serial sections of 10 μm thickness were cut using a microtome. Prior to staining, the sections were deparaffinized with xylene and rehydrated through a graded ethanol series. H&E staining was performed to visualize tissue morphology. For consistent evaluation, three representative cross-sectional slices from each sample were selected, and four distinct microscopic fields per slide were captured at 200× magnification. All images were obtained using an upright light microscope under identical imaging conditions.

### Enzyme-linked immunosorbent assay (ELISA)

To investigate the effects of PNS and NGR1 on UVB-triggered inflammation and oxidative stress, serum samples were collected from nude mice in the control, model, positive control, PNS-treated, and NGR1-treated groups. The concentrations of pro-inflammatory and anti-inflammatory cytokines—tumor necrosis factor-alpha (TNF-α), interleukin-1 beta (IL-1β), interleukin-6 (IL-6), and interleukin-10 (IL-10), were determined using ELISA kits (Jiangsu Enzyme-linked Immunosorbent Assay Industry Co., Ltd.; Batch Nos.: MM-013M1, MM-0040M1, MM-0163M1, MM-0176M1). Furthermore, markers of oxidative stress, including malondialdehyde (MDA), superoxide dismutase (SOD), total antioxidant capacity (T-AOC), and catalase (CAT), were analyzed with assay kits provided by Nanjing Jiancheng Bioengineering Institute (Batch Nos.: A003-1, A001-1, A015, A007-1), according to the manufacturer’s instructions.

### UVB irradiation

UVB irradiation of cells was carried out following previously described procedures [35]. Prior to exposure, cells were washed twice with 1× phosphate-buffered saline (PBS, Invitrogen) and then irradiated with UVB light at a dose of 20 mJ/cm^2^ (unless otherwise specified) using a UV Stratalinker 2400 instrument (Stratagene) equipped with UVB-emitting lamps. Control groups underwent identical handling but were not exposed to radiation (sham). The actual UVB intensity was regularly monitored and calibrated using a Goldilux UV meter fitted with a UVB sensor (Oriel Instruments) to ensure consistent dosing across experiments.

### Cell viability

To evaluate the cytotoxic impact of UVB radiation on HaCaT keratinocytes and examine the possible protective roles of PNS and NGR1, cell viability was assessed using the CCK-8 assay. Cells were seeded into 96-well plates at a density of 1 × 10^4^ cells per well and incubated for 24 h to allow attachment. Subsequently, cells were pretreated with increasing concentrations of PNS and NGR1 (0, 250, 500, and 1000 μM) for 24 h prior to UVB exposure. Following irradiation, the same concentrations of compounds were reapplied, and cells were cultured for an additional 24 h. In separate experiments, PNS and NGR1 were administered at a fixed concentration of 250 μM, with cell viability measured at various time points (0, 24, 48, 72, and 96 h). After treatment, 10 μL of CCK-8 reagent (Beyotime, Shanghai, China) was added to each well, and the plates were incubated for 4 h at 37 °C in a humidified 5% CO_2_ atmosphere. The generated formazan dye was quantified by measuring absorbance at 450 nm using a Tecan Infinite M1000 microplate reader (Tecan, Switzerland).

### Immunofluorescent staining

After UVB exposure, HaCaT and NAT10 knockdown HaCaT cells (2 × 10^5^ cells per well) were seeded onto cell culture inserts placed in 6-well plates and cultured for 24 h. Cells were then fixed with 4% paraformaldehyde for 15 min and permeabilized using PBS supplemented with 0.2% Triton X-100 for 20 min. To reduce non-specific binding, cells were blocked with 3% bovine serum albumin (BSA) for 30 min at room temperature. Primary antibodies against microtubule-associated protein 1A/1B light chain 3 (LC3, diluted 1:75), green fluorescent protein (GFP), and mCherry (diluted 1:1000) were applied, followed by overnight incubation at 4 °C. Following three washes, cells were incubated with appropriate fluorescent secondary antibodies conjugated to the respective host species. Nuclei were stained with DAPI (500 ng/mL) for 5 min, and fluorescence images were captured using an Olympus inverted fluorescence microscope.

### Western blot

Protein extraction was performed by washing cells with ice-cold PBS, followed by cell lysis in RIPA buffer (Thermo Fisher Scientific, Waltham, MA, USA) containing protease and phosphatase inhibitor cocktail (Thermo Fisher Scientific, Waltham, MA, USA). The lysates were subjected to sonication to achieve complete cell disruption and then centrifuged at 13,000×*g* for 20 min at 4 °C to pellet insoluble components. Protein concentration was determined using the Pierce BCA Protein Assay Kit (Thermo Fisher Scientific, Waltham, MA, USA). Following quantification, protein samples were normalized and denatured at 70 °C for 10 min. Proteins were then resolved by SDS–polyacrylamide gel electrophoresis and electro transferred onto PVDF membranes for immunoblot analysis. The following primary antibodies were employed:

- Anti-GAPDH (Proteintech, Cat#10494, 1:2000),
- Anti-β-actin (Bioss, bs-0061R, 1:2000),
- Anti-AKT1 (Servicebio, GB13011, 1:500),
- Anti-PI3K (Proteintech, Cat#27921, 1:500),
- Anti-mTOR (Proteintech, Cat#66888, 1:500),
- Anti-Bax (Proteintech, Cat#68111, 1:500),
- Anti-Bcl-2 (Proteintech, Cat#68103, 1:500),
- Anti-Caspase 3 (Proteintech, Cat#19677, 1:500),
- Anti-p62 (Abcam, ab207305, 1:1000),
- Anti-Beclin-1 (Abcam, ab207612, 1:1000),
- Anti-NAT10 (Abcam, ab194297, 1:2000),
- Anti-NBR1 (Abcam, ab55474, 1:1000),
- Anti-p-AKT1 (Bioss, bs-12456R, 1:500),
- Anti-p-mTOR (Bioss, bs-3495R, 1:500).

### Flow cytometry

Flow cytometry was conducted using a FACSCalibur flow cytometer (Becton Dickinson, USA). To assess apoptosis, 293 T cells transfected with NAT10-specific siRNA were irradiated with UVB at a dose of 20 mJ/cm^2^ and collected 24 h after exposure. In parallel, HaCaT cells were irradiated with UVB (20 mJ/cm^2^) and then incubated for 24 h in the presence or absence of PNS and NGR1. Following treatment, cells were harvested, washed twice with ice-cold PBS, and resuspended in 500 μL of 1× binding buffer. Apoptosis was analyzed by dual staining with annexin V and propidium iodide (PI), where early apoptotic cells were defined as annexin V-positive and PI-negative (AV+ /PI−), and late apoptotic or necrotic cells showed positive signals for both markers (AV+ /PI+). Flow cytometric data was collected and processed to determine the percentages of cells in different stages of apoptosis.

### RNA extraction and quantitative realtime-PCR (qPCR)

Total RNA was isolated using a Trizol Kit (G3013, Qiangen, China). The extracted RNA was then converted into cDNA through reverse transcription with the TUREscript 1st Strand cDNA Synthesis Kit (Aidlab, USA), adhering to the manufacturer’s instructions. The concentration of the resulting cDNA was quantified using a Nanodrop 2000 spectrophotometer (Thermo Scientific). The quantitative real-time polymerase chain reaction (qRT-PCR) analysis was carried out using the ABI Prism 7000 sequence detection system (provided by Thermo Fisher Scientific) in conjunction with the SYBR-Green master mix. The mRNA expression levels of the target genes—NAT10, were normalized to those of the β-actin housekeeping gene. Relative quantification of gene expression was calculated using the 2^−ΔΔCT^ method.

### Assessment of the NAT10 degradation pathway

Following 24 h of UVB irradiation, HaCaT cells were treated with either the proteasome inhibitor MG132 (10 μM) or the lysosomal inhibitor bafilomycin A1 (BafA1, 50 ng/mL) for 6 or 24 h, while a control group remained untreated. After incubation, total protein was extracted and subjected to immunoblotting to assess the expression levels of NAT10 and the loading control GAPDH, to determine the potential degradation pathway involved in NAT10 regulation.

### siRNA transfection

The following siRNAs were used: siRNA-ATG7 (sc-41447), siRNA-ATG5 (sc-41445), siRNA-NAT10 (sc-62660), and negative control siRNA (sc-37007), all purchased from Santa Cruz Biotechnology. Individual siRNAs were transfected into respective cell lines using an optimized siRNA transfection medium (sc-36868) and a dedicated siRNA transfection reagent (sc-29528), following the manufacturer’s recommended protocol.

### Utilization of NAT10 pull-down coupled with proteomic profiling

For NAT10 pull-down assays, the Universal Magnetic Beads Co-Immunoprecipitation (Co-IP) Kit (Proteintech, Cat#54002) was employed following the manufacturer’s protocol. Protein samples were resuspended in a lysis buffer containing 1% SDC, 100 mM Tris–HCl (pH 8.5), 10 mM TCEP, and 40 mM CAA. Denaturation, reduction, and alkylation were achieved by heating the mixture at 95 °C for 10 min. After centrifugation, the supernatant was diluted with an equal volume of ddH_2_O to reduce detergent concentration. Trypsin was added at a ratio of 1:50 (w/w, enzyme to protein) and digestion proceeded overnight at 37 °C. The reaction was terminated the following day by adjusting the pH to 6.0 with trifluoroacetic acid (TFA). Following a second centrifugation (12,000×*g*, 15 min), peptides in the supernatant were purified using a self-prepared SDB-RPS desalting column. Eluted peptides were vacuum-dried and stored at −20 °C until further analysis.

Sample analysis was performed using a timsTOF Pro mass spectrometer (Bruker Daltonics), which integrates trapped ion mobility spectrometry (TIMS) with quadrupole time-of-flight (Q-TOF) technology. The instrument was coupled online to an UltiMate 3000 RSLC nano liquid chromatography system (Thermo Scientific) and equipped with a Captive Spray nano electrospray ion source (Bruker Daltonics). Peptide mixtures were first loaded onto a C18 Trap column (75 µm × 2 cm, 3 µm particle size, 100 Å pore size, Thermo) and then separated on a reversed-phase C18 analytical column (75 µm × 15 cm, 1.7 µm particle size, 100 Å pore size, IonOpticks). Chromatographic separation was achieved using a gradient of solvent A (0.1% formic acid in water) and solvent B (0.1% formic acid in acetonitrile) at a flow rate of 300 nL/min. Data acquisition was carried out in diaPASEF mode, with the spray voltage set to 1500 V. Both MS and MS/MS spectra were acquired over an *m*/*z* range of 100–1700, while ion mobility measurements spanned a range of 0.6–1.6 Vs/cm^2^. Accumulation and ramp times were both set to 50 ms. The diaPASEF method was defined on the *m*/*z*-ion mobility plane using Bruker’s timsControl software. Collision energy was adjusted dynamically based on ion mobility, decreasing linearly from 59 eV at 1/K0 = 1.6 Vs/cm^2^ to 20 eV at 1/K0 = 0.6 Vs/cm^2^.

The DIA raw data were analyzed using DIA-NN (version 1.8.2 beta 11) in a library-free mode. The acquired spectral files were searched against the human protein sequence database downloaded from UniProt on August 7, 2024 (comprising 20,654 entries), without relying on an experimental spectral library. Default search settings were applied with specific modifications: in silico spectral prediction was enabled to generate a theoretical library; trypsin/P was selected as the cleavage enzyme, allowing up to two missed cleavages. Carbamidomethylation of cysteine residues was set as a fixed modification, while methionine oxidation and protein N-terminal acetylation were specified as variable modifications. Mass tolerance for precursor ions and MS1 scans were set to 15 ppm. Match-between-runs (MBR) and heuristic-based protein inference were activated to enhance detection accuracy. Peptide-spectrum matches were filtered to maintain a precursor-level false discovery rate (FDR) of 1%. Protein quantification was performed using the MaxLFQ algorithm for intensity normalization across samples.

Protein quantification was performed based on the ‘pg_matrix.tsv’ file generated from DIA-NN search results. Subsequent bioinformatics processing was conducted using the R programming environment. Missing values were imputed using a random Gaussian model centered around the detection limit of the mass spectrometer, with parameters set to a mean downshift of 1.8 standard deviations and a spread of 0.25 standard deviations to reflect low-abundance signal characteristics. For data filtering, only proteins with at least 50% valid values in at least one experimental group were retained for further analysis. Differentially expressed proteins (DEPs) were identified by combining statistical significance from two-sample Student’s *t* tests (*P* < 0.05) with predefined fold change cutoffs. Functional enrichment analysis was carried out using multiple annotation resources, including Gene Ontology (GO), KEGG, EggNOG, Pfam, and UniProt-based subcellular localization information. Enriched biological terms and pathways were determined using Fisher’s exact test to assess overrepresentation within the significantly regulated protein sets. Furthermore, protein–protein interaction(PPI) networks were constructed and analyzed using the STRING database to explore functional associations among the identified proteins.

### Co-IP

Cells were washed twice with ice-cold PBS, harvested on ice using a cell scraper, and centrifuged at 1000 rpm for 5 min at 4 °C. After removal of the supernatant, the cell pellet was resuspended in cold Co-IP lysis buffer (Proteintech, Cat#PR20037) by gentle pipetting until complete dispersion of cell clumps was achieved. The lysate was incubated on ice for 30 min to ensure full lysis, followed by centrifugation at 13,000 rpm for 15 min at 4 °C. The supernatant was then carefully collected and transferred to a new pre-chilled tube for subsequent immunoprecipitation or protein analysis.

Each reaction tube was supplemented with 20 μL of magnetic beads and an optimized quantity of immunoprecipitation antibody dilutions predetermined for maximum specificity and efficiency. The mixtures were rotated at 4 °C for 4 h to facilitate antibody-bead binding. After incubation, tubes were placed on a magnetic rack to separate the beads, and the supernatant was carefully removed. The beads were then resuspended in pre-chilled cell lysis buffer and incubated overnight at 4 °C to allow target antigen capture. Following this incubation, unbound proteins were removed by discarding the supernatant, and the bead-bound complexes were washed 2–3 times with PBS to reduce nonspecific interactions. Finally, the immunoprecipitated complexes were eluted by resuspending the beads in 25 μL of 5× Laemmli sample buffer, heated at 95 °C for 5–10 min to denature proteins, and subjected to immunoblot analysis.

### Analysis of network pharmacology

The saponin profile of PNS was analyzed using UHPLC-Q-Orbitrap-MS/MS. Following identification, the SMILES notations for each saponin were obtained from the PubChem database. These molecular structures were then submitted to the Swiss Target Prediction platform (http://www.swisstargetprediction.ch/) to predict potential protein targets, considering only those with a prediction probability greater than zero. In addition, experimentally reported targets from existing literature were incorporated to generate a comprehensive and refined list of saponin-associated targets through data integration.

A comprehensive search was performed using the keywords “skin sunburn injury,” “skin inflammation,” and “skin cancer” across three public databases: GeneCards (https://www.genecards.org/), OMIM (https://www.omim.org/), and DrugBank (https://www.drugbank.ca/), to collect genes associated with skin sunburn-related conditions. The resulting target lists from each database were then integrated using Draw Venn (http://bioinformatics.psb.ugent.be/webtools/Venn/) to construct a Venn diagram, enabling the identification of overlapping genes that may represent potential therapeutic targets for skin sunburn injury.

PPI networks were constructed by importing the common gene targets identified in both datasets into the STRING database (http://string-db.org), with species limited to *Homo sapiens* and a minimum interaction confidence score of 0.9. The resulting interaction data were then visualized and further analyzed using Cytoscape 3.7.2 (https://www.cytoscape.org/) to build a core target network, enabling comprehensive evaluation of topological features and identification of potentially critical nodes within the network.

To explore the influence of PNS on gene functions and signaling pathways involved in UVB-induced skin sunburn injury, functional enrichment analyses were conducted using the DAVID database (https://david.ncifcrf.gov/), including GO and KEGG pathway annotation. The resulting data were visualized using R software (version 3.4.1), with emphasis on the top 10 enriched GO terms and top 30 KEGG pathways, applying significance thresholds of FDR < 0.05 and * *P* < 0.05. Subsequently, ginsenoside–target–pathway (G–T–P) interaction networks were constructed to map the relationships among active compounds, their molecular targets, and the associated biological pathways, providing a comprehensive view of potential mechanisms underlying PNS activity.

### Molecular docking study

The high-resolution crystal structure of the target protein was retrieved from the Protein Data Bank (RCSB, https://www.rcsb.org). Using PyMOL 2.5.2, non-protein components such as water molecules and cofactors were removed to prepare the protein structure, which was then saved in PDB format. The three-dimensional(3D) molecular structure of the ligand was obtained from the PubChem database and converted into either MOL2 or PDB format using Open Babel 3.1.1 for compatibility with docking software. Molecular docking simulations were performed in AutoDock 4.2.6 by integrating the processed protein and ligand structures. The conformation exhibiting the lowest binding energy was selected as the optimal docking pose. A docking score below 0 kcal/mol indicates a favorable interaction, while values less than −5 kcal/mol suggest strong binding potential. The resulting binding modes and interaction patterns were visualized and analyzed using PyMOL.

### Statistical analysis

Data are presented as mean ± standard deviation (S.D.). To assess significant differences between groups, one-way analysis of variance (ANOVA) was conducted, followed by post-hoc multiple comparisons using Student’s *t* test. All statistical evaluations were carried out using SPSS software (version 16.0, SPSS Inc., Chicago, IL, USA). A * *P* value less than 0.05 was considered statistically significant.

## Results

### Analysis of saponin profile in PNS

**Fig. 1:**
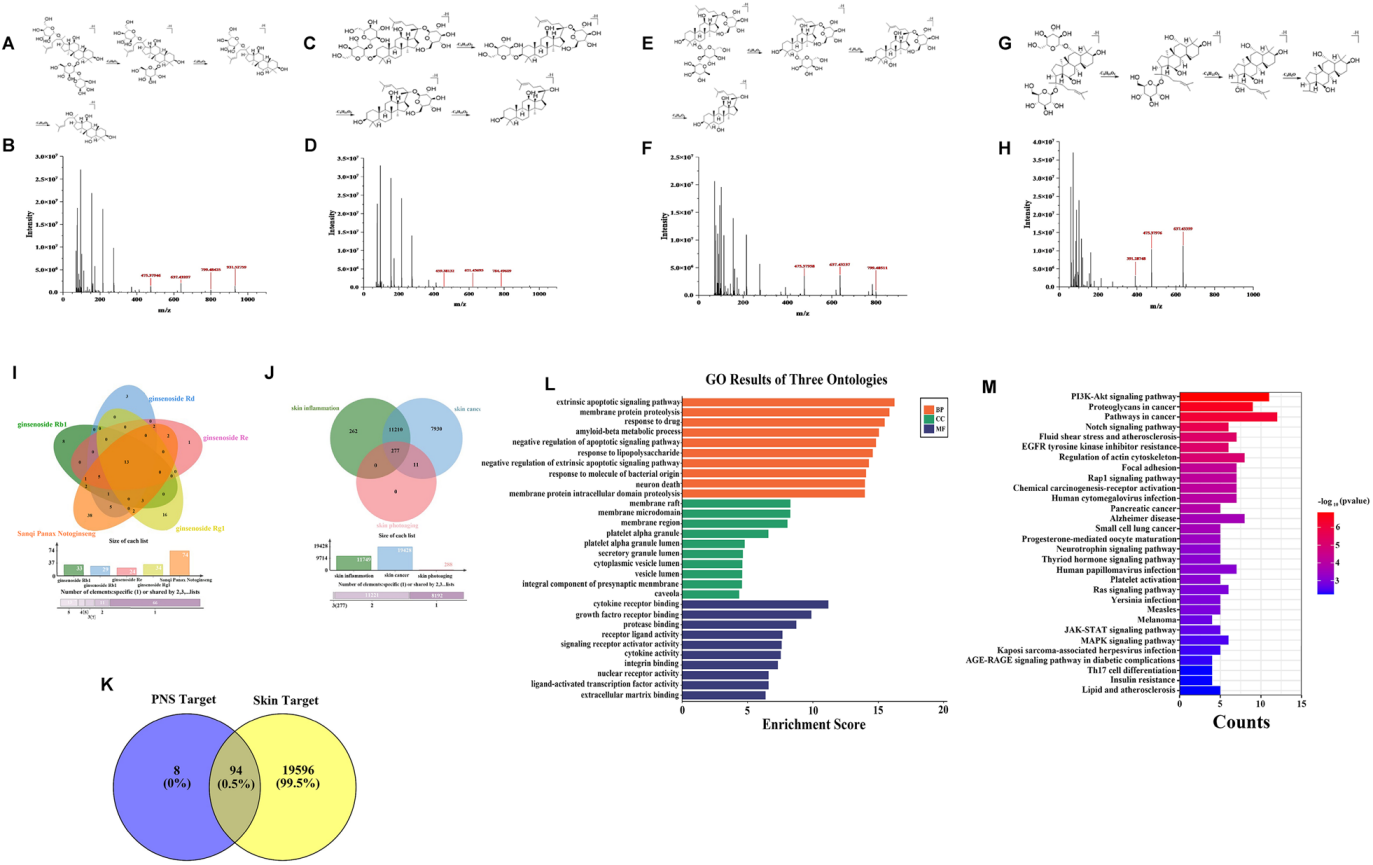
Chemical component analysis and network pharmacology analysis of PNS. **A**,**B** Fragmentation pathways of NGR1 and its MS spectrum in negative ion mode. **C**,**D** Fragmentation pathways of Ginsenoside Rd and its MS spectrum in negative ion mode. **E**,**F** Fragmentation pathways of Ginsenoside Re and its MS in negative ion mode. **G**,**H** Fragmentation pathways of Ginsenoside Rg1 and its MS in negative ion mode. **I** The targets of peripheral nervous system overlap with Ginsenoside Rb1, Ginsenoside Rg1, Ginsenoside Rd, NGR1, and Ginsenoside Re. **J** The overlapping targets of UVB damage encompass skin cancer, cutaneous inflammation, and skin sunburn injury. **K** The Venn diagram demonstrates the convergence of PNS targets associated with UVB-induced skin sunburn injury. **L** The top 10 BP, CC, and molecular function terms identified in the GO analysis are visually represented by bars colored orange, green, and blue respectively. **M** The bar graph displays the top 30 pathways determined through KEGG enrichment analysis

The chemical composition of PNS was systematically characterized using UHPLC-Q-Orbitrap-MS/MS, with the total ion chromatogram presented (Supplementary Fig. 1A). By analyzing high-resolution mass spectra, tandem fragmentation patterns, and comparing data with the PNS database and published literature, a total of 16 saponin compounds were confidently identified. Detailed information on these identified constituents is summarized (Supplementary Table 1 and Supplementary Table 2). The combined content of five major components, NGR1, Ginsenoside Rb1, Ginsenoside Rg1, Ginsenoside Rd, and Ginsenoside Re, was quantified following the procedures outlined in the 2020 edition of the Chinese Pharmacopoeia, with the reference chromatogram displayed in (Supplementary Fig. 1B and 1C). Quantitative determination of these 5 saponins was further conducted via UHPLC-Q-Trap-MS/MS (Supplementary Fig. 1D and 1E), revealing their total abundance to be 84.13%, confirming their status as principal bioactive constituents in PNS; hence, they were selected for subsequent network pharmacology investigations. Fragmentation pathways were proposed based on UHPLC-Q-Orbitrap-MS/MS spectral data, enabling the structural elucidation of 4 key saponins: NGR1, Ginsenoside Rd, Ginsenoside Re, and Ginsenoside Rg1 (Fig. 1A–H). Additionally, the individual concentrations of all 16 ginsenosides were determined using HPLC, with results provided (Supplementary Table 7).

### PPI analysis

To identify the SMILES identifiers of the five key saponin compounds in PNS, namely NGR1, Ginsenoside Rb1, Ginsenoside Rg1, Ginsenoside Rd, and Ginsenoside Re, a systematic search was conducted in the PubChem database. The resulting SMILES strings were then submitted to the Swiss Target Prediction platform for target profiling, which yielded 198 predicted molecular targets (Fig. 1I). In parallel, a comprehensive search across the GeneCards, OMIM, and DrugBank databases enabled the collection of 31,465 gene targets associated with UVB-induced skin sunburn injury (Fig. 1J). All retrieved gene targets were standardized and processed for redundancy using UniProt, resulting in 19,689 unique candidate targets. Subsequently, an intersection analysis between the 198 compound-related targets and the 19,689 disease-associated targets identified 94 overlapping genes, which represent potential therapeutic targets of PNS in mitigating UVB-induced skin sunburn injury (Fig. 1K).

A total of 94 shared targets were uploaded to the STRING database, generating a PPI network composed of 82 nodes and 1015 edges (Supplementary Fig. 1F). The refined network was constructed by filtering nodes with a degree value greater than or equal to the median (≥ 23), resulting in a densely interconnected topology. Key hub proteins identified within the network include AKT1, IL6, Bax, Bcl-2, CXCL8, FN1, MAPK8, IL1B, HSP90AA1, and GSK3B, among others. These proteins ranked among the top 11 central nodes based on their Degree, Betweenness Centrality (BC), and Closeness Centrality (CC) values (Supplementary Table 3), highlighting their critical roles in the network architecture. These highly connected targets are thus considered pivotal candidates through which PNS may exert protective effects against UVB-induced skin sunburn injury.

### Performing GO and KEGG pathway enrichment analysis

GO functional enrichment and KEGG pathway analyses were performed using the DAVID 6.8 platform to explore the biological roles of the 94 overlapping targets linked to ginsenosides and skin sunburn injury. The top 10 enriched terms in each of the three GO categories (biological process, cellular component, and molecular function) are presented in bar charts (Fig. 1L). A bubble plot further displays the top 20 significantly enriched KEGG pathways, illustrating how PNS may exert protective effects against UVB-induced skin damage through multi-pathway modulation (Fig. 1M). The KEGG analysis revealed that total saponins might be involved in 30 signaling pathways relevant to UVB-induced skin sunburn injury. Key pathways include PI3K-AKT, Notch, Rap1, Neurotrophins, thyroid hormone, Ras, JAK-STAT, and MAPK signaling. Among these, the PI3K-AKT, Notch, and Rap1 pathways demonstrate particularly prominent enrichment. These critical pathways involve 16 central targets identified in the PPI network, such as GF, cytokines, ECM, ITGA, ITGB, AKT, HSP90, Bcl-2, and NF-κB. Based on both the number and relative proportion of target genes associated with each pathway, the PI3K-AKT signaling pathway stands out as the most likely mechanism underlying the repair of UVB-induced skin sunburn injury by PNS (Supplementary Table 4). This suggests that activation or regulation of the PI3K-AKT pathway may play a pivotal role in the therapeutic effects of PNS.

The GO and KEGG enrichment analyses indicate that PNS may exert protective effects against UVB-induced skin sunburn injury through the activation of the PI3K-AKT and mTOR signaling pathways. To illustrate these pathways, the “Path view” package in R was utilized to generate integrated pathway maps. These results imply that the therapeutic mechanism of PNS in alleviating UVB-mediated skin damage likely involves the regulation of the PI3K-AKT-mTOR signaling cascade, highlighting this axis as a key contributor to its pharmacological activity.

### Ginsenoside–target–pathway (G–T–P) network analysis

To better understand the complex relationships between ginsenosides, their molecular targets, and associated biological pathways in PNS, a comprehensive ginsenoside–target–pathway (G–T–P) network was established (Supplementary Fig. 1G). A detailed summary of the 16 saponins in PNS and their involvement in pathways related to UVB-induced protein damage is provided (Supplementary Table 5; Supplementary Fig. 1H). The network consists of 65 nodes: 5 ginsenosides, 50 target proteins, and 10 enriched signaling pathways, interconnected by multiple regulatory associations. In this visualization, green nodes represent potential target proteins, purple nodes denote ginsenosides, and yellow nodes indicate signaling pathways; the edges reflect their functional interactions. Node size and opacity are proportional to their degree of connectivity, reflecting greater biological relevance. The G–T–P network reveals that each ginsenoside interacts with multiple targets across various pathways (Supplementary Fig. 1G and 1H), supporting a multi-target, multi-pathway mode of action for PNS in alleviating UVB-induced skin sunburn injury. Notably, key proteins such as Rela, Jun, Stat3, IL2, Fos, and Bcl-2 exhibit high connectivity (degree = 11, 11, 11, 10, 10, and 10, respectively), underscoring their central roles in mediating the therapeutic effects of PNS. Furthermore, the prominent involvement of the PI3K-AKT pathway highlights its critical contribution to the protective mechanism of PNS against such disease.

### PNS treatment significantly attenuates UVB-induced skin sunburn injury in a nude mouse model

**Fig. 2:**
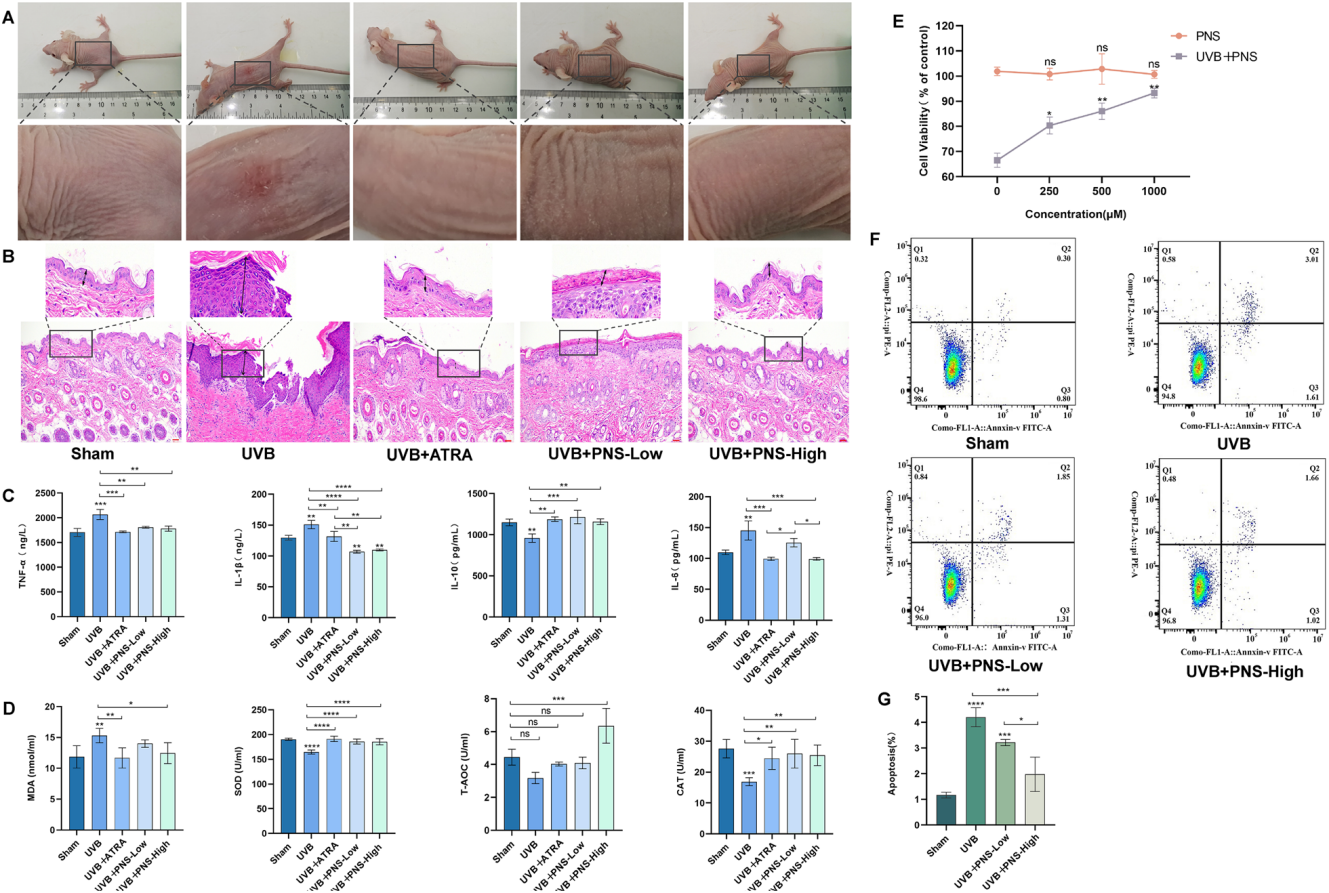
The administration of PNS effectively alleviates UVB-induced sunburn injury in nude mice and enhances the viability of HaCaT cells when exposed to UVB irradiation, while also inhibiting apoptosis in these cells under UVB irradiation. **A** The photographs depict mouse skin samples from various experimental groups following exposure to UVB radiation. **B** Microscopic images of skin sections stained with H&E were acquired from the control group, UVB irradiation group, ATRT positive drug-treated group, and PNS-treated group. **C** The levels of TNF-α, IL-1β, IL-6, and IL-10 in the serum of UVB-irradiated nude mice were determined through ELISA analysis following PNS treatment. **D** ELISA analysis showed that after PNS treatment, the levels of MDA, SOD, T-AOC, and CAT in the serum of UVB-irradiated nude mice were determined. **E** Viability diagram of HaCaT cells after treatment with different concentrations of PNS (0, 250, 500, and 1000 μM) and viability chart of HaCaT cells exposed to UVB radiation and treated with varying concentrations (0, 250, 500, and 1000 μM) of PNS. **F**,**G** The impact of PNS on apoptosis of HaCaT cells was assessed by flow cytometry using the Annexin V-FITC/PI method to determine cell apoptosis rate. The experiments were conducted using a minimum of three biologically independent samples (*n* ≥ 3). Error bars represent the mean ± standard deviation (S.D.) values (**C**, **D**). Statistical significance was determined using Student’s *t* test with N.S. non-significant differences, * *P* < 0.05, ** *P* < 0.01, *** *P* < 0.001, **** *P* < 0.0001 denoting different levels of significance

H&E staining results demonstrated significant skin sunburn injury in nude mice after UVB irradiation, marked by increased epidermal thickness and visible signs of sunburn on the dorsal skin. In contrast, administration of PNS markedly reduced epidermal hyperplasia and effectively mitigated UVB-induced cutaneous injury (Fig. 2A, B). PNS treatment significantly lowered the levels of pro-inflammatory cytokines, TNF-α, IL-1β, and IL-6, in UVB-exposed animals, while promoting the expression of the anti-inflammatory cytokine IL-10 (Fig. 2C). Moreover, PNS enhanced the activity of key antioxidant enzymes, including SOD, T-AOC, and CAT, in irradiated mice, and simultaneously reduced MDA levels, indicating attenuated oxidative stress (Fig. 2D). These results indicate that PNS exhibits a protective effect against such disease in nude mice by suppressing inflammation and reinforcing the endogenous antioxidant defense system.

### PNS treatment improves the viability of HaCaT cells exposed to UVB radiation

HaCaT cells treated with various concentrations of PNS (0, 250, 500, and 1000 μM) showed no significant cytotoxicity, with cell viability remaining above 95% (Fig. 2E). In contrast to the control group, exposure to 20 mJ/cm^2^ of UVB radiation induced substantial cell death, reducing cell viability to approximately 50%. However, post-irradiation treatment with increasing concentrations of PNS markedly improved cell survival in a dose-dependent manner (Fig. 2E). Additionally, when HaCaT cells were exposed to PNS over different time intervals (0, 24, 48, 72, and 96 h), no notable cytotoxic effects were observed, as viability consistently exceeded 95% (Supplementary Fig. 2B). These results indicate that PNS effectively promotes the survival of HaCaT cells under UVB stress, supporting its protective role against radiation-induced cellular damage.

### PNS treatment reduces UVB-induced apoptosis in HaCaT cells

The impact of PNS on UVB-induced apoptosis in HaCaT cells was assessed by flow cytometry. Following UVB irradiation, the apoptotic cell population reached 4.62% in the HaCaT cell line. Following treatment with 250 μM and 500 μM PNS, the proportion of apoptotic cells decreased to 3.63% and 1.86%, respectively, demonstrating a significant reduction in cell apoptosis (Fig. 2F, ). These results indicate that PNS effectively suppresses UVB-triggered apoptotic processes in HaCaT cells in a concentration-dependent manner.

### PNS suppresses the activation of the PI3K/AKT/mTOR signaling pathway induced by UVB irradiation

**Fig. 3:**
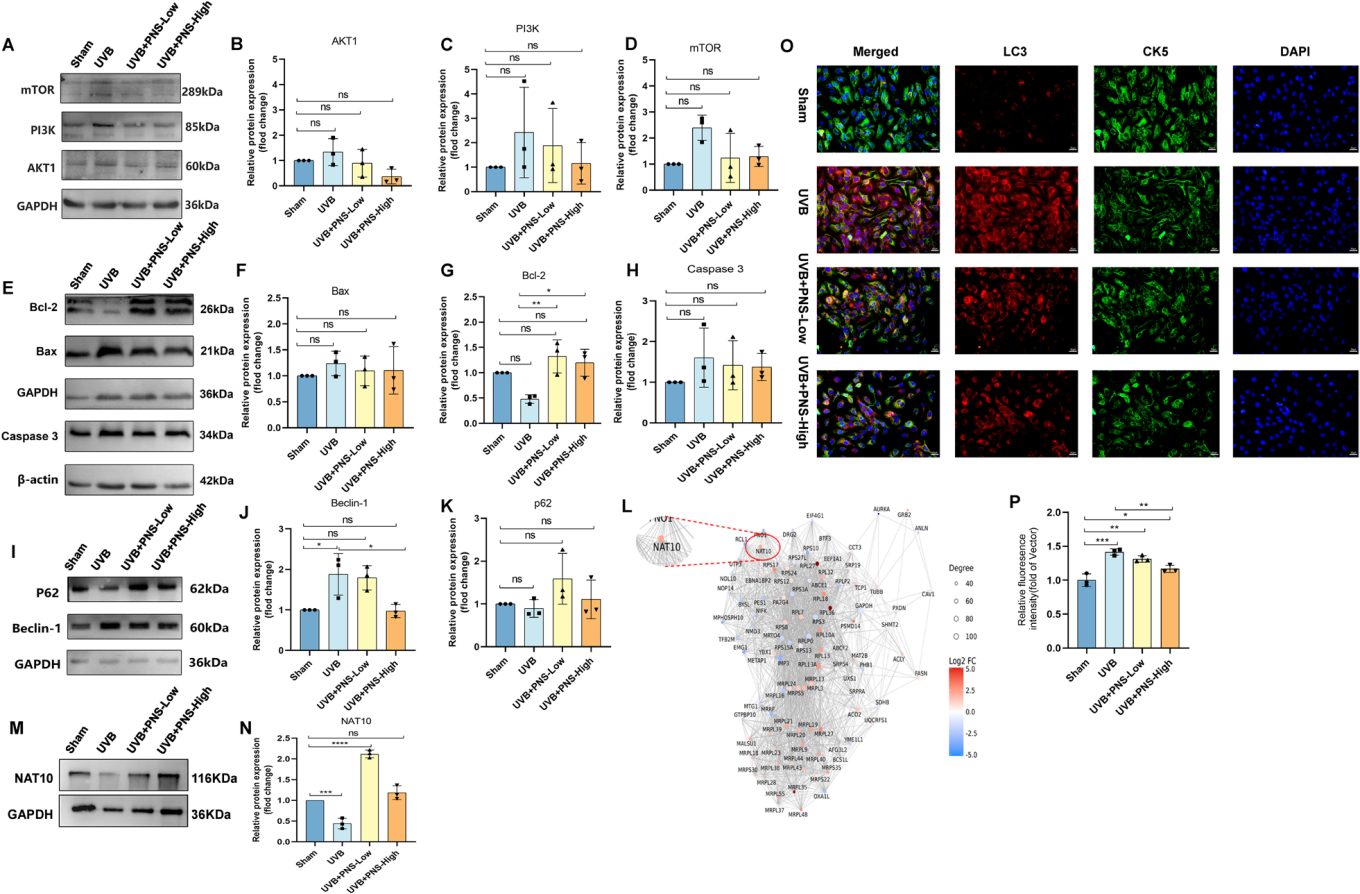
The activation of the PI3K/AKT/mTOR signaling pathway is inhibited by PNS upon UVB irradiation, while it also regulates the expression of apoptosis and autophagy proteins. **A**–**D** The effect of UVB and PNS on the expression of PI3K/AKT/mTOR pathway proteins. **E**–**H** The effect of UVB and PNS on the expression of apoptosis-related proteins Bax, Bcl-2, and Caspase 3. **I**,**G**,**K** The effect of UVB and PNS on autophagy in HaCaT cells. **L** PPI network diagrams will be generated for the proteins identified in the sham and UVB groups, based on their degree values. **M**,**N** Treatment with concentrations of 250 μM and 500 μM PNS, followed by cell irradiation, a significant upregulation in the expression level of NAT10. **O**,**P** Immunofluorescence detection of LC3 in HaCaT cells (×200). The expression levels of target genes, including mTOR, PI3K, AKT1, Bax, Bcl-2, Caspase 3, p62 and Beclin-1 were normalized to the expression levels of housekeeping proteins GAPDH and β-actin. The experiments were conducted using a minimum of three biologically independent samples. Error bars represent the mean ± S.D. **A**–**C**,**G** Statistical significance was determined by Student’s *t* test with N.S. non-significant differences, * *P* < 0.05, ** *P* < 0.01, *** *P* < 0.001, **** *P* < 0.0001 denoting different levels of significance

Network pharmacology analysis indicates that PNS may exert therapeutic effects against UVB-induced skin sunburn injury by modulating multiple signaling pathways, such as PI3K-AKT, proteoglycans in cancer, pathways in cancer, Notch, and Rap1 signaling. Among these, the PI3K/AKT/mTOR pathway showed prominent enrichment in KEGG pathway analysis, suggesting its central role in the underlying mechanism. UVB irradiation led to a marked increase in the expression of key proteins, PI3K, AKT1, and mTOR, in HaCaT cells. However, treatment with 250 μM and 500 μM PNS significantly reduced the expression levels of these proteins following UVB exposure (Fig. 3A–D). Furthermore, using siRNA-control and siRNA-NAT10 cell models, we investigated the molecular regulatory role of NGR1, a major bioactive monomer in PNS. Western blot results demonstrated that both NGR1 and NAT10 influence the phosphorylation levels of AKT1 (p-AKT1) and mTOR (p-mTOR) (Fig. 7J–L), indicating their involvement in pathway regulation. These findings collectively suggest that PNS attenuates UVB-induced activation of the PI3K/AKT/mTOR signaling cascade, thereby contributing to its protective effect on skin cells.

### PNS modulates the expression of apoptosis-related proteins following UVB exposure

Network pharmacology predictions indicate that PNS may contribute to the repair of UVB-induced skin damage by modulating apoptosis-related pathways. Experimental data confirm that PNS exerts a protective effect by suppressing UVB-triggered apoptosis in HaCaT cells. Further analysis showed that treatment with PNS (250 and 500 μM) significantly increases the expression of the anti-apoptotic protein Bcl-2, while reducing the levels of pro-apoptotic proteins Bax and Caspase 3 in UVB-exposed HaCaT cells (Fig. 3E–H). These results indicate that PNS regulates the balance of apoptotic proteins under UVB stress, thereby inhibiting programmed cell death and promoting the recovery of UVB-damaged skin tissue.

### PNS modulates the levels of autophagy-associated proteins following UVB exposure

Network pharmacology predictions suggest that PNS may facilitate the recovery from UVB-induced skin injury by modulating autophagy. This study explores the effect of PNS on autophagic activity in HaCaT cells after UVB irradiation. The expression of critical autophagy markers, LC3-II, Beclin-1, and p62, was assessed via Western blotting and immunofluorescence staining. The findings revealed that treatment with high-dose PNS significantly upregulated p62 protein levels while markedly downregulating Beclin-1 expression in UVB-exposed cells (Fig. 3I–K). Immunofluorescence imaging further confirmed that high-dose PNS substantially decreased LC3 puncta formation, indicating reduced LC3-II activation (Fig. 3O, P). These findings suggest that PNS suppresses excessive autophagy in response to UVB radiation by altering the expression of critical autophagy markers, thereby protecting skin cells and facilitating the recovery from UVB-induced sunburn injury.

### The downregulation of RNA acetylase NAT10 induced by UVB can be effectively reversed in a dose-dependent manner by PNS

Based on proteomic data, we constructed PPI networks for the sham and UVB groups, revealing strong interconnectivity and functional clustering of NAT10-associated proteins under both conditions (Fig. 3L). The consistent presence of NAT10 within these networks highlights its potential regulatory role in the cellular response to UVB-induced skin sunburn injury. Further experiments in HaCaT cells showed a significant downregulation of NAT10 protein expression following UVB irradiation. Notably, treatment with 250 and 500 μM PNS markedly restored NAT10 protein levels in a dose-dependent manner (Fig. 3M, N). Collectively, these findings demonstrate that PNS effectively counteracts UVB-induced suppression of the acetyltransferase NAT10, supporting its potential involvement in maintaining NAT10-mediated molecular functions during UVB-induced skin sunburn injury.

### NGR1 exhibits the most favorable binding profile for alleviating UVB-induced skin sunburn injury

**Fig. 4:**
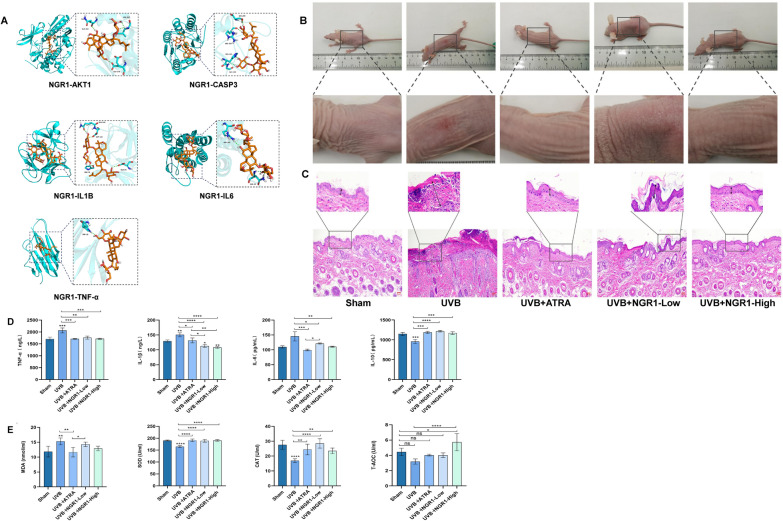
The administration of NGR1 effectively alleviates UVB-induced sunburn injury in nude mice. **A** Visualization map of docking between NGR1 and target molecules. **B** The photographs depict mouse skin samples from various experimental groups following exposure to UVB radiation. **C** Microscopic images of skin sections stained with H&E were acquired from the control group, UVB irradiation group, ATRT positive drug-treated group, and NGR1-treated group. **D** The levels of TNF-α, IL-1β, IL-6, and IL-10 in the serum of UVB-irradiated nude mice were determined through ELISA analysis following NGR1 treatment. **E** ELISA analysis showed that after NGR1 treatment, the levels of MDA, SOD, T-AOC, and CAT in the serum of UVB-irradiated nude mice were determined. The experiments were conducted using a minimum of three biologically independent samples (*n* ≥ 3). Error bars represent the mean ± standard deviation (S.D.) values (**D**, **E**). Statistical significance was determined using Student’s *t* test with N.S. non-significant differences, * *P* < 0.05, ** *P* < 0.01, *** *P* < 0.001, **** *P* < 0.0001 denoting different levels of significance

To investigate the binding interactions between potential targets and bioactive compounds, molecular docking was performed on the five core targets with the highest degree values in the PPI network along with their corresponding chemical components (Supplementary Table 6). All docking complexes displayed binding energies below −5 kJ/mol, accompanied by the formation of hydrogen bonds between ligands and receptors, indicating strong and stable molecular interactions. Visualization using PyMOL revealed that NGR1 achieved the most favorable docking outcomes with the top-ranked core targets, as evidenced by high docking RMSD values and robust binding affinities—each below −5.0 kJ/mol—with clear hydrogen bond formations observed (Fig. 4A). Detailed analysis indicated that NGR1 exhibits the strongest interaction capability among all tested components during PNS-mediated protection against UVB-induced skin sunburn injury, suggesting its central role in the pharmacological activity of PNS. In the structural diagrams, green dashed lines represent hydrogen bonds, orange-yellow regions depict the chemical structure of the compound, and blue areas highlight the active binding sites on the target proteins.

### NGR1 treatment significantly attenuates UVB-induced skin sunburn injury in a nude mouse model

Histopathological analysis via H&E staining showed pronounced sunburn injury in the skin of UVB-irradiated nude mice, as evidenced by significant epidermal thickening and visible erythema on the dorsal surface. In contrast, treatment with NGR1 markedly attenuated epidermal hyperplasia and alleviated UVB-triggered skin sunburn injury (Fig. 4B, C). Moreover, NGR1 administration effectively downregulated the levels of major pro-inflammatory cytokines—TNF-α, IL-1β, and IL-6—in irradiated animals, while upregulating the expression of the anti-inflammatory cytokine IL-10 (Fig. 4D). Additionally, NGR1 boosted the enzymatic activities of key antioxidant defenses, including SOD, T-AOC, and CAT, in UVB-exposed mice, and simultaneously decreased MDA levels, a recognized indicator of oxidative stress (Fig. 4E). Taken together, these findings indicate that NGR1 protects against UVB-induced skin sunburn injury in nude mice by modulating inflammatory responses and enhancing antioxidant capacity.

### NGR1 treatment markedly improves the viability of HaCaT cells following UVB irradiation

**Fig. 5:**
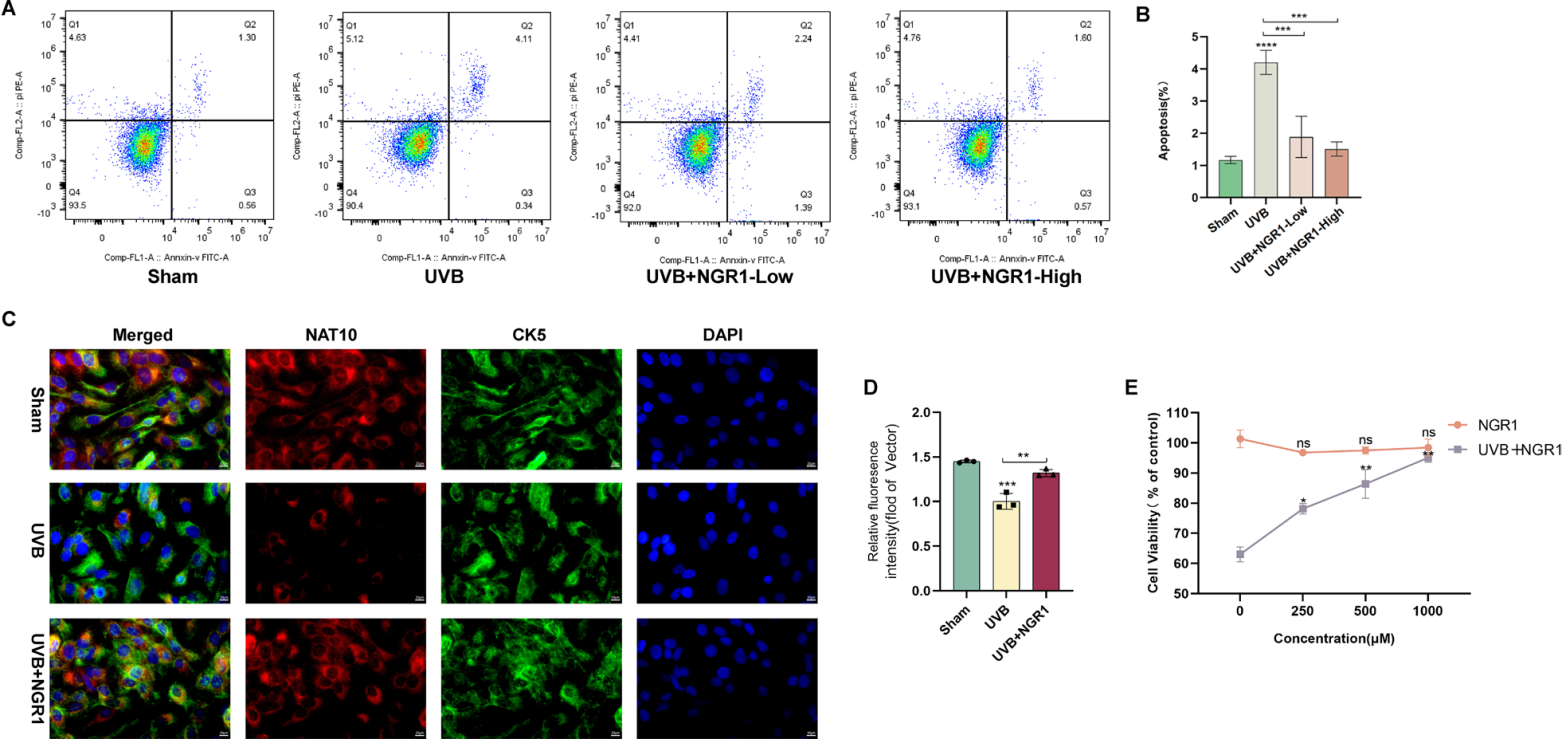
The presence of NGR1 enhances the survival rate of HaCaT cells under UVB irradiation, while inhibiting apoptosis of these cells under UVB irradiation and regulating the degradation of the RNA acetylase NAT10. **A** Viability diagram of HaCaT cells after treatment with different concentrations of NGR1 (0, 250, 500, and 1000 μM) and viability chart of HaCaT cells exposed to UVB radiation and treated with varying concentrations (0, 250, 500, and 1000 μM) of NGR1. **B**,**C** The impact of NGR1 on apoptosis of HaCaT cells was assessed by flow cytometry using the annexin V-FITC/PI method to determine cell apoptosis rate. **D**,**E** Immunofluorescence detection of NAT10 in HaCaT cells (×400). All data were performed on *n* ≥ 3 biologically independent samples. Error bars are shown as mean ± S.D. **A**,**B** *P* values by Student’s *t* test with N.S. non-significant differences, * *P* < 0.05, ** *P* < 0.01, *** *P* < 0.001, **** *P* < 0.0001

HaCaT cells exposed to different concentrations of NGR1 (0, 250, 500, and 1000 μM) showed no significant cytotoxicity, maintaining cell viability above 95% across all treatment groups. In comparison, irradiation with 20 mJ/cm^2^ UVB markedly decreased cell survival, resulting in approximately 50% viability compared to the untreated control. However, following UVB irradiation, treatment with increasing concentrations of NGR1 led to a significant, dose-dependent recovery in cell viability (Fig. 5E). Additionally, when cells were exposed to NGR1 over different time periods (0, 24, 48, 72, and 96 h), no substantial toxicity was observed, as viability remained above 95% across all time points (Supplementary Fig. 2C). These findings demonstrate that NGR1 effectively promotes the survival of HaCaT cells subjected to UVB radiation without inducing cytotoxicity.

### NGR1 treatment suppresses UVB-induced apoptosis in HaCaT cells and modulates the expression of RNA acetyltransferase NAT10

The effect of NGR1 on apoptosis in UVB-exposed HaCaT cells was assessed via flow cytometry. Analysis revealed that UVB irradiation alone induced an apoptotic rate of 4.62%. Upon treatment with 250 μM and 500 μM NGR1, the proportion of apoptotic cells dropped to 3.09% and 1.30%, respectively, demonstrating a dose-dependent suppression of apoptosis (Fig. 5A, B). Immunofluorescence staining further showed that high-concentration NGR1 markedly reduced the expression of the RNA acetyltransferase NAT10 in HaCaT cells following UVB exposure (Fig. 5C, D). These findings suggest that NGR1 exerts anti-apoptotic effects in UVB-irradiated HaCaT cells and modulates the UVB-induced upregulation of NAT10 by promoting its downregulation.

### Quantitative proteomic profiling of different groups after NAT10 affinity enrichment

**Fig. 6:**
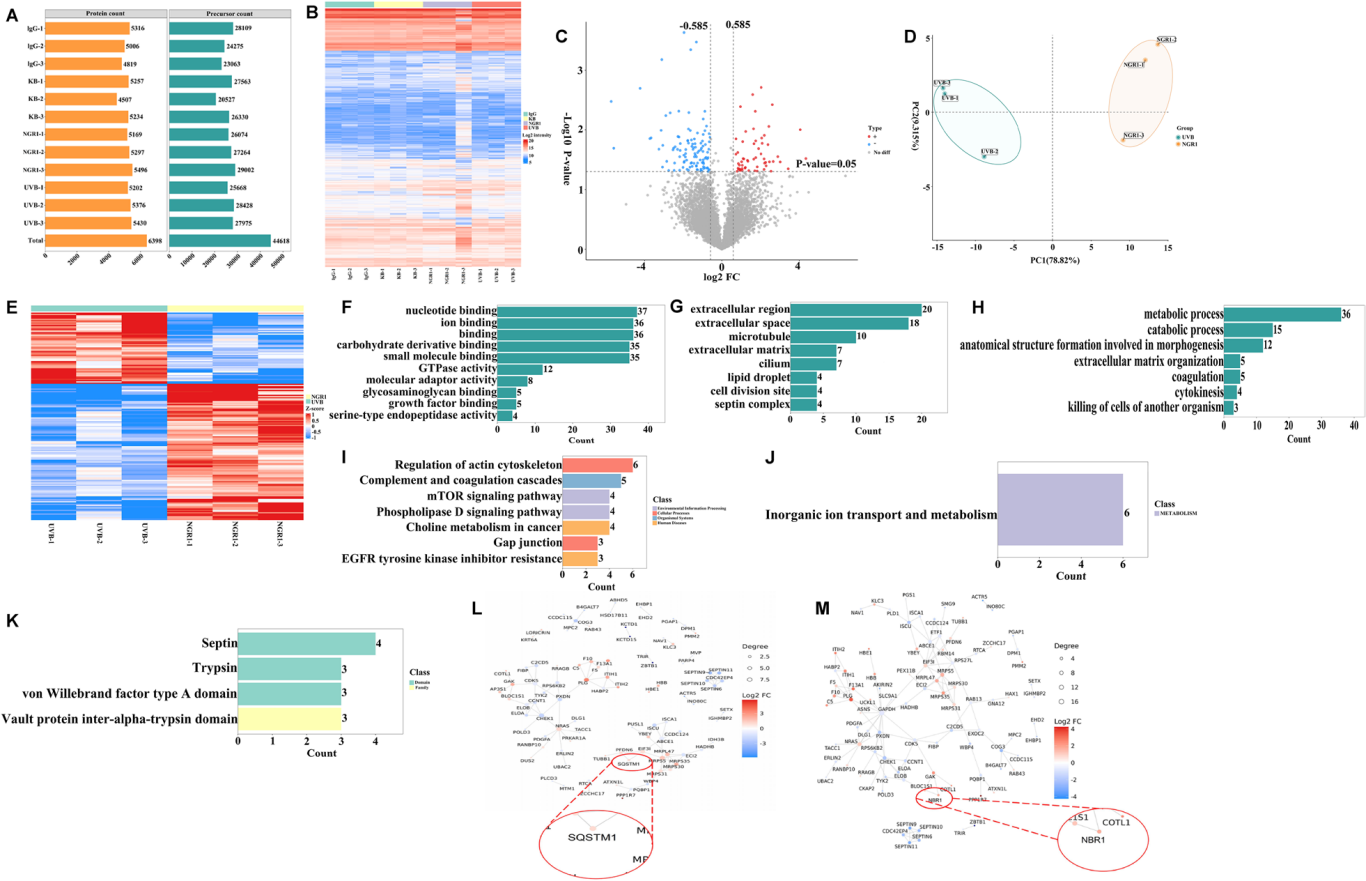
The study involved conducting differential proteomics analysis and in vitro validation on different experimental groups after the knockdown of NAT10. **A** The sample overview images obtained from protein quantification across different experimental groups were comparatively analyzed, with a comprehensive statistical evaluation of the number of peptides and proteins identified and quantified in each individual sample. **B** A heatmap is generated based on quantitative correlation coefficients. **C** The volcano plot depicts the distinct protein expression patterns between the UVB group and the NGR1 group. **D** The PCA plot demonstrates the differential protein expression between the UVB group and NGR1 group. **E** The quantitative heatmap illustrates the variation in protein expression between the UVB group and NGR1 group. **F**–**H** The bubble chart depicts the enrichment of GO terms for proteins identified in the UVB and NGR1 groups, encompassing BP, CC, and molecular functions . **I** The bar chart depicts the KEGG pathway annotations of proteins identified in the UVB and NGR1 groups. **J** The bar chart illustrates the COG annotations of proteins identified in the UVB and NGR1 groups. **K** The bar chart depicts the Pfam annotations of proteins identified in the UVB and NGR1 groups. **L** The protein interaction network diagrams will be generated based on the log_2_ fold change (FC) values for the proteins identified in both the UVB and NGR1 groups. **M** The protein interaction network diagrams will be generated for the proteins identified in the UVB and NGR1 groups, based on their degree values

To investigate proteomic differences across experimental groups, NAT10 pull-down assays were performed on the UVB group treated with vehicle, and UVB-induced group treated with NGR1 (250 μM), followed by in-depth quantitative proteomic profiling. A total of 44,618 unique peptides, corresponding to 6398 individual proteins, were confidently identified through integrated analysis of three biological replicates from each group (Fig. 6A). The quantitative reproducibility between replicate samples was assessed by calculating Pearson correlation coefficients for all sample pairs. A heatmap visualizing these correlation values was then constructed to illustrate the overall consistency and reliability of the proteomic measurements across the dataset (Fig. 6B).

### Comparative proteomic profiling of different groups after NAT10 affinity purification

Peptide signal intensities were extracted by searching mass spectrometry raw data against a protein sequence database, enabling the quantification of corresponding proteins. After normalizing the quantitative data, protein levels were compared across different samples. A comparative analysis between the UVB and NGR1-treated groups was conducted, with results visualized through volcano plots to highlight differentially expressed proteins (Fig. 6C), principal component analysis (PCA) to assess sample distribution and grouping trends (Fig. 6D), and hierarchical clustering heatmaps to display variations in protein expression profiles (Fig. 6E). This integrative approach led to the identification of several key autophagy-associated selective adaptors or proteins, p62, NBR1, ATG5, MTOR, and MAP1LC3B2, among the significantly altered proteins. These findings imply that NGR1 may mitigate UVB-induced downregulation of RNA acetyltransferase NAT10 by activating or modulating autophagy-related pathways.

### Proteome annotation and functional enrichment analysis were conducted across different groups following NAT10 affinity purification

GO annotation was carried out for all identified proteins to categorize their BP (Fig. 6F), CC (Fig. 6G), and molecular function(Fig. 6H). Furthermore, KEGG pathway enrichment analysis revealed significant involvement of the identified proteins in the mTOR signaling pathway key regulator of autophagy (Fig. 6I). This supports the hypothesis that NGR1 modulates UVB-induced downregulation of the acetyltransferase NAT10 via autophagy-related mechanisms. To explore evolutionary and functional classifications, COG (Clusters of Orthologous Groups) analysis was performed on the proteomes from both the UVB and NGR1-treated groups (Fig. 6J), revealing a prominent association with “Inorganic ion transport and metabolism.” Additionally, Pfam domain annotation was applied to identify conserved protein domains across the detected proteome, with the distribution of major domains illustrated (Fig. 6K). These comprehensive annotations provide functional context for the differentially expressed proteins and reinforce the link between NGR1’s protective effects and autophagy regulation under UVB stress.

### PPI network analysis following NAT10 affinity purification was performed in distinct experimental groups

The STRING database was utilized to analyze differentially expressed proteins between the UVB and NGR1-treated groups, enabling the construction of PPI networks. Two distinct network maps were generated based on the log_2_ fold change (log_2_ FC) and Degree values, respectively (Fig. 6L, M). In these networks, each node represents a differentially expressed protein, and the connecting edges indicate known or predicted functional interactions. Node colors reflect the magnitude of differential expressions or associated scoring metrics, while node size corresponds to the degree of connectivity, highlighting hub proteins within the network. Key interacting proteins identified in these networks include PLG, MRPS35, GAK, MRPS5, EIF3I, and F13A1, suggesting their potential involvement in the molecular mechanisms modulated by NGR1 under UVB exposure.

### NAT10 deficiency enhances the susceptibility of cells to UVB-induced apoptosis

The influence of NAT10 on apoptosis induced by UVB radiation in 293 T cells was assessed via flow cytometry. Analysis revealed that silencing NAT10 using siRNA led to an increase in the apoptotic rate to 6.00%, compared to 3.64% in the control group (Fig. 8A). These results suggest that downregulation of the RNA acetyltransferase NAT10 sensitizes 293 T cells to UVB-induced apoptosis, underscoring its essential role in regulating cellular responses to UVB-mediated skin sunburn injury.

### UVB-induced downregulation of NAT10 is mediated via autophagic degradation

**Fig. 7:**
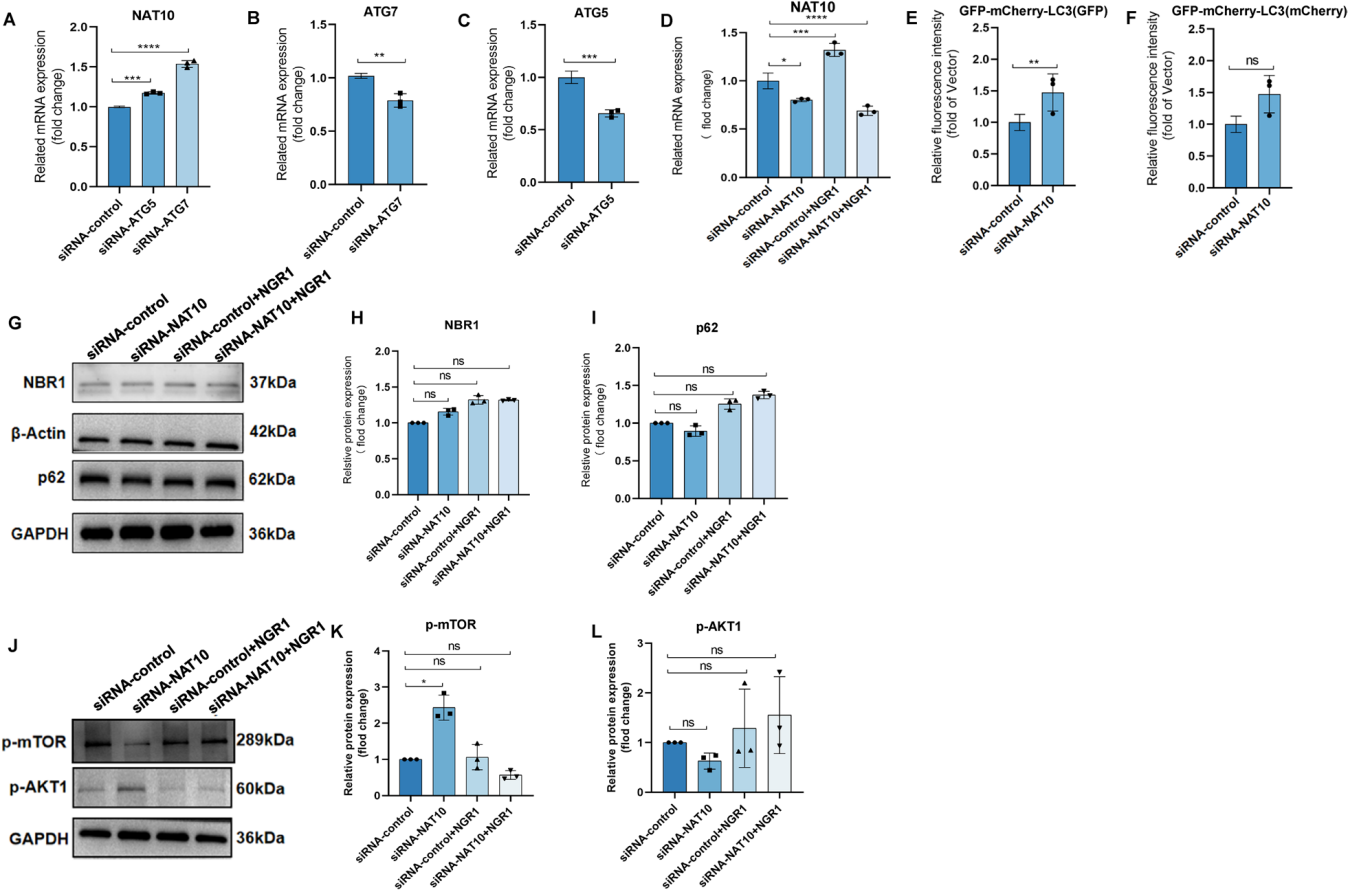
NGR1 regulates NAT10 expression through the autophagic pathway and functions in the activated state of autophagy. **A** After ATG7 and ATG5 knockdown, the expression of NAT10 significantly increased compared to the control group. **B** According to qPCR results, the expression level of ATG7 was significantly downregulated after gene knockdown, confirming the success of the knockdown. **C** According to qPCR results, the expression level of ATG5 was significantly downregulated after gene knockdown, confirming the success of the knockdown. **D** In the control group and NAT10 knockdown group, treatment with DMSO and NGR1, respectively, revealed that NGR1 upregulates the expression of NAT10 in the control group. **E** Expression levels of GFP in immunofluorescence. **F** Expression levels of mCheryy in immunofluorescence. **G**–**I** In the absence of UVB-induced autophagy activation, NGR1 does not regulate p62 and NBR1. **J**–**L** NGR1 significantly modulates the protein expression levels of p-AKT1 and p-mTOR in both siRNA-control and siRNA-NAT10 HaCaT cells. The error bars represent the mean ± S.D. **A**–**F**,**H** Statistical significance was determined using Student’s *t* test with N.S. non-significant differences, * *P* < 0.05, ** *P* < 0.01, *** *P* < 0.001, **** *P* < 0.0001 indicating different levels of significance

**Fig. 8:**
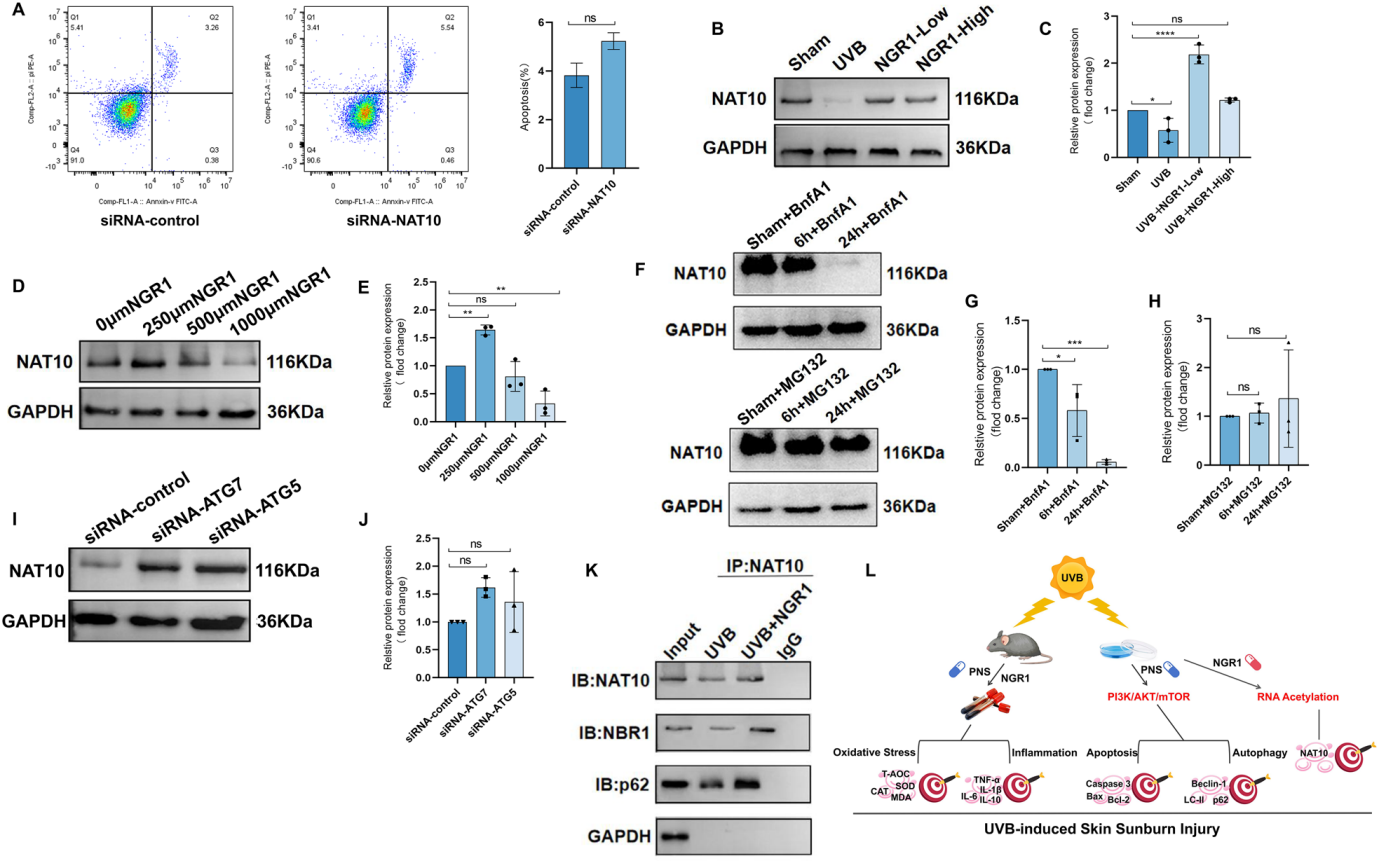
UVB radiation downregulates NAT10 expression through autophagic pathways. Notably, NGR1 effectively reverses the dose-dependent suppression of the RNA acetyltransferase NAT10 induced by UVB, mediated by the autophagy selective receptors p62 and NBR1. **A** The effect of NAT10 on apoptosis in UVB-irradiated 293T cells was analyzed using flow cytometry. **B**,**C** Following treatment with 250 μM and 500 μM NGR1 and subsequent UVB irradiation, a significant increase in NAT10 protein expression was observed. **D**,**E** Administration of NGR1 at varying concentrations (0, 250, 500, and 1000 μM) resulted in a significant, dose-dependent modulation of NAT10 protein expression in HaCaT cells. **F**–**H** The proteasome inhibitor MG132 had no effect on the UVB-induced downregulation of NAT10, whereas the lysosomal function inhibitor BfnA1 significantly suppressed this downregulation. **I**,**J** Genetic inhibition of autophagy, achieved by deleting essential autophagy-related genes ATG5 or ATG7 in HaCaT cells, effectively abrogated the UVB-induced downregulation of NAT10. **K** HaCaT cells were subjected to co-immunoprecipitation using control species-matched IgG and anti-NAT10 antibodies, followed by immunoblotting to detect NBR1, p62, and NAT10. **L** The molecular mechanism underlying the therapeutic effects of PNS and NGR1 on UVB-induced skin sunburn injury. The error bars represent the mean ± S.D. **A**–**C**,**I** Statistical significance was determined using Student’s *t* test with N.S. non-significant differences, * *P* < 0.05, ** *P* < 0.01, *** *P* < 0.001, **** *P* < 0.0001 indicating different levels of significance

Preliminary experiments were performed to examine the impact of UVB irradiation on NAT10 expression in keratinocytes. UVB markedly downregulated NAT10 levels in HaCaT cells. To elucidate the underlying molecular mechanism of this decrease, the potential roles of the two primary protein degradation systems: the ubiquitin–proteasome pathway and the autophagy-lysosome pathway were investigated. Treatment with MG132, a proteasomal inhibitor, failed to reverse the UVB-induced decrease in NAT10, indicating minimal proteasomal involvement. In contrast, BafA1, an inhibitor of lysosomal acidification and autophagic flux, effectively prevented the downregulation of NAT10 following UVB exposure (Fig. 8F–H). To further validate the role of autophagy,HaCaT cell lines with knockdown either of essential autophagy genes ATG5 and ATG7 were generated. Western blot and qPCR analyses confirmed that loss of these genes significantly alleviated the UVB-triggered decline in NAT10 protein levels (Fig. 7A–C, 8I, and 8J), reinforcing the notion that autophagy is central to NAT10 regulation under UVB stress (Fig. 7G, I). In functional rescue experiments, treatment with NGR1 or DMSO led to a pronounced increase in NAT10 expression in control cells (siRNA-control), whereas NAT10 levels remained substantially reduced in cells transfected with siRNA targeting NAT10 (siRNA-NAT10) (Fig. 7D). Furthermore, immunofluorescence assays revealed diminished autophagic activity upon NAT10 knockdown. Using a tandem fluorescent mCherry-GFP-LC3 reporter system, a marked accumulation of GFP signal—indicative of impaired lysosomal degradation due to blocked autophagic flux—in NAT10-deficient cells was observed, while mCherry fluorescence remained stable (Fig. 7E, F; Supplementary Fig. 2A). These findings offer clear evidence that loss of NAT10 impairs autophagosome maturation and clearance. Collectively, the results robustly indicate that the reduction of NAT10 following UVB exposure is predominantly regulated via the autophagy-lysosome degradation pathway.

### NGR1 effectively restores UVB-induced downregulation of the RNA acetyltransferase NAT10 in a dose-dependent manner

Exposure to UVB radiation led to a marked reduction in NAT10 protein levels in HaCaT cells. However, treatment with NGR1 at concentrations of 250 and 500 μM significantly restored NAT10 expression (Fig. 8B, C). When cells were treated with increasing doses of NGR1 (0, 250, 500, and 1000 μM), a clear dose-dependent upregulation of NAT10 protein was observed (Fig. 8D, E), indicating a concentration-responsive effect mediated by NGR1. These findings demonstrate that NGR1 effectively reverses the suppression of RNA acetyltransferase NAT10 caused by UVB irradiation in a dosage-dependent manner.

### NGR1 facilitates the autophagic clearance of NAT10 under UVB stress via recruitment of NBR1 and p62

To investigate the mechanism responsible for UVB-induced downregulation of NAT10, this study focused on the autophagy selective receptors p62 and NBR1, guided by proteomic profiling. Initial results showed that NGR1 treatment did not significantly alter p62 or NBR1 levels in non-irradiated HaCaT cells (Fig. 7G–I). Following UVB exposure, cells were treated with 250 μM or 1000 μM NGR1. Co-IP analysis demonstrated direct interactions between NAT10 and both p62 and NBR1, suggesting that NGR1 facilitates the autophagic degradation of NAT10 by enhancing its association with these autophagy selective adaptors (Fig. 8K). These findings indicate that NGR1 promotes NAT10 clearance under UVB stress through a receptor-mediated autophagy pathway involving p62 and NBR1.

## Discussion

Owing to ongoing environmental changes, ozone depletion has begun, resulting in the development of ozone holes that weaken the Earth’s shielding capacity against UVB radiation [36]. Therefore, increased levels of UVB radiation reaching the surface are linked to a higher risk of skin disorders and impaired immune function [37]. Understanding the molecular mechanisms involved in UVB-induced skin injury, as well as potential protective strategies, is therefore of both scientific importance and practical relevance.

 UVB radiation exposure can cause significant damage to the dermal extracellular matrix, particularly affecting structural proteins such as collagen and elastin. Histopathological features of this damage include severe epidermal injury, aberrant keratinocyte proliferation, and localized melanocyte hyperplasia [38–40]. UVB radiation induces adverse morphological changes in skin tissue, leading to impaired skin barrier function, increased epidermal thickness, wrinkle formation, and loss of skin elasticity [39, 41]. Key histological alterations include uneven epidermal thickening, pronounced hyperkeratosis in the stratum corneum, flattening of the dermo-epidermal junction, and reduction or disappearance of dermal papillae. Within the dermis, inflammatory cell infiltration is commonly observed, along with disrupted elastic fiber architecture, manifested as fiber fragmentation or clumping. Collagen fibers also undergo structural and quantitative changes, while elastin accumulates abnormally [42]. Moreover, collagen exhibits progressive degeneration, characterized by fiber breakage and disorganized alignment. Microvasculature becomes tortuous and dilated, often surrounded by inflammatory infiltrates, and focal melanocyte proliferation is evident [42]. Skin photodamage is a multifaceted biological process that involves several interconnected mechanisms, including apoptosis, oxidative stress, upregulation of matrix metalloproteinases (MMPs), and impaired regulation of cellular autophagy [43–45].

Panax notoginseng is rich in a class of highly bioactive constituents referred to as PNS, which have been extensively utilized in clinical settings [46, 47]. The primary components of PNS are NGR1, Ginsenoside Rg1, Ginsenoside Rb1, Ginsenoside Rd, and Ginsenoside Re. A growing body of evidence has demonstrated the potent anti-inflammatory properties of Ginsenoside Rb1, Ginsenoside Rg1, and NGR1 [48–50]. In addition, Ginsenoside Rg1, Ginsenoside Re, Ginsenoside Rb1, and Ginsenoside Rd have been widely investigated for their roles in anti-aging, anticancer, and immune-regulating effects. Nevertheless, despite their promising pharmacological potential, the development and application of PNS, particularly its specific constituent NGR1, remains limited and requires further in-depth research.

NGR1, a key monomeric constituent of PNS, is characterized by a clearly defined chemical structure and well-understood physicochemical properties. In contrast to the heterogeneous composition of total PNS, the use of NGR1 as a purified single compound offers substantial benefits in terms of manufacturing consistency and quality assurance. Its high degree of purity allows for precise dosage control, thereby improving reliability, safety, and therapeutic effectiveness in clinical applications. Moreover, while PNS may trigger unpredictable allergic responses due to its complex formulation, the side effect profile of NGR1 is more predictable and easier to manage. Current evidence indicates that NGR1 exhibits no notable toxicity at clinically relevant doses. Numerous studies have established that NGR1 plays a protective role against UVB-induced skin sunburn injury through diverse molecular pathways, including suppression of inflammation, scavenging of reactive oxygen species, enhancement of DNA repair mechanisms, and preservation of dermal fibroblast function.

The PI3K/AKT/mTOR signaling pathway plays a pivotal role in regulating key cellular processes such as cell survival, apoptosis, and autophagy, and has been strongly implicated in the initiation and advancement of multiple cancer types [51]. PI3K acts as an intracellular lipid kinase, while AKT functions as a serine/threonine-specific protein kinase. Upon activation by growth factor receptors, PI3K modulates the conformational state of AKT, initiating downstream signaling events that influence critical cellular processes. This activation cascade subsequently leads to the phosphorylation of pro-apoptotic factors such as Bad and Caspase-9, thereby regulating programmed cell death [52, 53]. UVB radiation has been shown to activate the PI3K/AKT pathway, which in turn promotes mTOR activity, a key suppressor of autophagy, resulting in reduced autophagic flux [54]. Under normal conditions, UVRAGs (UV radiation resistance-associated genes) play stimulatory roles in the process of the autophagosome formation; however, its downregulation inhibits autophagy initiation [55]. UVB exposure alters the transcriptional expression of several autophagy-related regulators, including AMPK, Sesn2, TSC2, and UVRAGs, disrupting the balance of autophagic activity within cells. These findings highlight the complex interplay between UVB-induced signaling and autophagy regulation through key molecular nodes in the PI3K/AKT/mTOR network.

Utilizing network pharmacology and molecular docking approaches, this study identified NGR1 as a pivotal bioactive compound with therapeutic potential. Comprehensive binding energy evaluations revealed that NGR1 plays a central role in mitigating UVB-induced skin sunburn injury through regulatory mechanisms involving cellular repair pathways. In general, UVB radiation triggers autophagy, a process modulated by multiple signaling cascades activated in response to UV exposure. Autophagy is a highly regulated dynamic process; its initiation involves the integration of various upstream signals converging on a key regulatory complex, mammalian target of rapamycin complex 1 (mTORC1) [52, 56]. This complex consists of core components including mTOR, Raptor, GβL/mLST8, PRAS40, and DEPTOR, with mTOR serving as the central kinase governing autophagic activity. Activation of autophagy is marked by increased levels of LC3-II, which promotes autophagosome formation and subsequent fusion with lysosomes, enabling the clearance of damaged cellular components. PNS treatment reduced the expression of PI3K, AKT, and mTOR proteins, suggesting an inhibitory effect on the PI3K/AKT/mTOR signaling cascade. These observations imply that PNS may enhance autophagy through the suppression of mTORC1 activity, thus playing a protective role in mitigating cellular damage caused by UVB radiation.

PPI network was constructed to map the core active components of PNS and their shared molecular targets associated with UVB-induced skin sunburn injury. Functional annotation via GO enrichment and KEGG pathway analyses revealed the biological functions and signaling networks associated with the identified key targets. To experimentally verify the predicted mechanisms, both in vitro and in vivo studies were conducted, which confirmed that PNS exerts its protective effects largely by inhibiting the PI3K/AKT/mTOR signaling pathway. The findings showed that PNS markedly downregulates the protein expression of PI3K, AKT, and mTOR, demonstrating a potent suppressive action on this central regulatory cascade. Additionally, the pharmacological profile of PNS was explored to identify a safe and effective therapeutic strategy for UVB-induced skin sunburn injury. Notably, no significant cytotoxicity was detected in keratinocytes following PNS treatment, supporting its favorable safety profile. Protein expression changes were used as key markers of UVB-induced cellular damage. The data revealed that PNS not only markedly reduces UVB-triggered apoptotic cell death but also modulates the expression of autophagy-related proteins, including p62, Beclin-1, and LC3-II, suggesting a regulatory role in autophagic processes induced by UVB exposure. Given its proven efficacy in various disease models, PNS emerges as a promising candidate for the prevention and treatment of UVB-mediated skin sunburn injury.

RNA acetylation, particularly the ac^4^C modification, represents a recently identified and functionally significant type of RNA chemical alteration, although its underlying mechanisms and regulatory elements are still being investigated. In contrast to the more extensively studied m^6^A modification, research on ac^4^C remains in its early stages, with limited knowledge about its regulatory factors. To date, only one primary catalytic enzyme has been associated with this modification, NAT10 [57]. NAT10 plays a central role in mediating ac^4^C formation on RNA molecules, contributing to the regulation of RNA stability, translation efficiency, and other post-transcriptional processes. Dysregulation or abnormal expression of NAT10 has been implicated in the development and progression of multiple pathological conditions, particularly cancer [58, 59]. Given its pivotal enzymatic function, targeting NAT10, either through modulation of its expression or inhibition of its activity, holds potential as a promising therapeutic approach for the intervention and management of diseases linked to aberrant RNA acetylation.

This study highlights several innovative findings regarding the use of PNS and its active constituent NGR1 in mitigating UVB-induced skin sunburn injury. PNS demonstrates potent antioxidant activity by effectively neutralizing UVB-generated reactive oxygen species and suppresses the release of pro-inflammatory cytokines, thereby reducing oxidative stress and inflammatory responses associated with sunburn. Moreover, we propose that the protective mechanism of PNS involves modulation of the PI3K/AKT/mTOR signaling pathway, which in turn attenuates excessive apoptosis and restores balanced autophagy—critical cellular processes implicated in UVB-induced skin sunburn injury. To enhance the robustness and reproducibility of these findings, the experimental design integrated both in vitro cell-based assays and in vivo animal models, supported by multiple molecular and histological assessment parameters. This comprehensive approach not only strengthens the scientific credibility of the results but also provides a translational framework for the development of other natural compounds as potential dermato-protective agents. Additionally, this work explores the mechanism underlying NAT10 degradation under UVB stress, revealing that NGR1 facilitates the autophagic clearance of NAT10 by promoting its interaction with the autophagy selective adaptors p62 and NBR1. All of them uncover a previously unrecognized regulatory axis involving NGR1-mediated selective autophagy in the context of UVB-induced skin sunburn injury.

In the absence of UVB-induced autophagy activation, the impact of NGR1 on p62 and NBR1 in HaCaT cells treated with siRNA-control or siRNA-NAT10 was investigated. The findings suggest that under conditions where autophagy is not activated, NGR1 does not significantly regulate p62 and NBR1. This may be attributed to the mechanism of action of NGR1, which appears to function not prior to autophagy initiation but rather during the stage of autophagy activation leading to the formation of autolysosomes. These findings offer novel perspectives on the molecular mechanisms underlying skin cell responses to UVB radiation and highlight potential therapeutic targets for the prevention and management of UVB-induced skin sunburn injury. By systematically investigating NAT10’s involvement in such disease , this study aims to clarify its functional significance and lay a solid groundwork for future translational and clinical developments. Owing to their natural origin, PNS and NGR1 exhibit high biocompatibility and low toxicity, contributing to their strong potential for commercial application. These attributes highlight their value as innovative candidates in the evolution of modern dermatological and skincare therapeutics. The limitations in pathway selection. Further exploration revealed that, in addition to the existing research pathways, the NF-κB [58] and Notch signaling pathways [21,22] may play crucial roles in regulating UVB-induced skin sunburn injury by NGR1. To address the current research gaps, our laboratory plans to conduct systematic experiments to elucidate the potential mechanisms of these signaling pathways.

In conclusion, both PNS and NGR1 demonstrate significant efficacy in reducing UVB-induced apoptosis and autophagy in cellular models. The protective action of PNS is partly attributed to its regulation of the PI3K/AKT/mTOR signaling cascade, whereas NGR1 primarily functions by targeting the RNA acetyltransferase NAT10, influencing its stability and activity. Nevertheless, whether additional molecular mechanisms are involved in their protective roles against UVB-induced skin sunburn injury remains to be fully elucidated and warrants further research. Given their potent biological activities and natural origin, PNS and NGR1 hold considerable promise as therapeutic candidates for managing chronic skin inflammation and potentially preventing UVB-driven skin sunburn injury, highlighting their broad scientific and clinical potential.

## Conclusion

The results demonstrate that PNS effectively scavenges reactive oxygen species induced by UVB exposure and inhibits the secretion of inflammatory mediators, thereby alleviating skin sunburn injury associated with photodermatitis. This study highlights that the protective effects of PNS are largely mediated through modulation of the PI3K/AKT/mTOR signaling cascade, along with the suppression of excessive apoptosis and dysregulated autophagy, both of which are critical in mitigating such disease. To ensure robustness and reproducibility, both in vivo animal models and in vitro cellular systems supported by multiple analytical endpoints and provided a comprehensive framework that may inform the future development of natural product-based therapies. Regarding the specific compound NGR1, particular emphasis was placed on its interaction with the RNA acetyltransferase NAT10 (Fig. 8L). Experimental evidence confirmed that NGR1 promotes the downregulation of NAT10 under UVB stress conditions. Furthermore, we identified that NGR1 facilitates the autophagic degradation of NAT10 by engaging the selective autophagy selective receptors p62 and NBR1, revealing a novel mechanism underlying its regulatory function. Due to their natural origin, PNS and NGR1 exhibit high biocompatibility, minimal cytotoxicity, and strong therapeutic potential, making them promising candidates for dermatological applications. These attributes underscore their significance as innovative components in the formulation of next-generation skincare agents.

## Supplementary Information

**Additional file 1. Supplementary Figure 1: Comprehensive analysis and pharmacological network of PNS in UVB-induced skin sunburn injury.** (A) TIC of qualitative analysis for PNS . (B) TIC of reference standards for PNS (1: NGR1; 2: Ginsenoside Rg1; 3: Ginsenoside Re; 4: Ginsenoside Rb1; 5: Ginsenoside Rd). (C) Representative TIC of test samples analyzed in negative ion mode. (D, E) UHPLC-Q-Trap-MS/MS analysis of PNS components (1-5 as labeled) in negative ion mode, including fragmentation pathways and corresponding mass spectra for NGR1. (F) PPI network of shared targets between PNS and UVB-induced skin sunburn injury. (G) Key pathways and target network of PNS in treating UVB-induced skin sunburn injury. (H) Component-target network diagram illustrating interactions between 15 PNS saponins and their molecular targets in UVB-induced skin sunburn injury. **Supplementary Figure 2: Functional analysis of NAT10 and saponin effects on HaCaT cell viability.** (A) Immunofluorescence imaging of GFP and mCherry expression in HaCaT cells transfected with siRNA-control or siRNA-NAT10. (B) Time-dependent cell viability assay of HaCaT cells treated with 250 μM PNS at 0, 24, 48, 72, and 96 hours. (C) Cell viability of HaCaT cells following treatment with 250 μM NGR1 across the same time intervals (0–96 h). Data represents mean±S.D. from three independent experiments. Statistical significance was determined using Student’s t-test with N.S. non-significant differences, * P<0.05, ** P<0.01 indicating different levels of significance.

Additional file 2.

## Data Availability

No datasets were generated or analysed during the current study.

## Abbreviations

AKT1: AKT Serine/Threonine Kinase 1
ATRA: All-trans Retinoic Acid
ATG5: Autophagy-related 5
ATG7: Autophagy-related 7
Bax: Bcl-2-associated X Protein
Bcl-2: B-cell Lymphoma 2
BafA1: Bafilomycin A1
Beclin-1: Beclin 1 Coiled-coil Domain Autophagy Regulator
CAT: Catalase Micrococcus Lysodeikticus
Caspase 3: Cysteine-aspartic Proteases 3
GAPDH: Glyceraldehyde-3-phosphate Dehydrogenase
H&E: Hematoxylin and Eosin
IL-1β: Interleukin-1β
IL-6: Interleukin-6
IL-10: Interleukin-10
LC3: Microtubule-associated Protein Light Chain 3
LC-MS/MS: Liquid Chromatography-Tandem Mass Spectrometry
mTOR: Mammalian Target of Rapamycin
NAT10: N4-acetyl transferase 10
NGR1: Notoginsenoside R1
PNS: Panax Notoginseng Saponins
PI3K: Phosphatidylinositol-3-kinase
p62: Sequestosome 1
SOD: Superoxide Dismutase
T-AOC: Total Antioxidant Capacity
UHPLC-Q-Orbitrap-MS/MS: Ultra-high-performance Liquid Chromatography Coupled with Quadrupole Orbitrap Mass Spectrometry
UVB: Ultraviolet B Radiation
