# Supplementary figures and images for: Notoginsenoside R1 mitigates UVB-induced skin sunburn injury through modulation of N4-acetylcytidine and autophagy
Source: Chin Med. 2025 Dec 18;20:216. doi: 10.1186/s13020-025-01270-3 (PMC12713273; doi:10.1186/s13020-025-01270-3)

## Slide 1
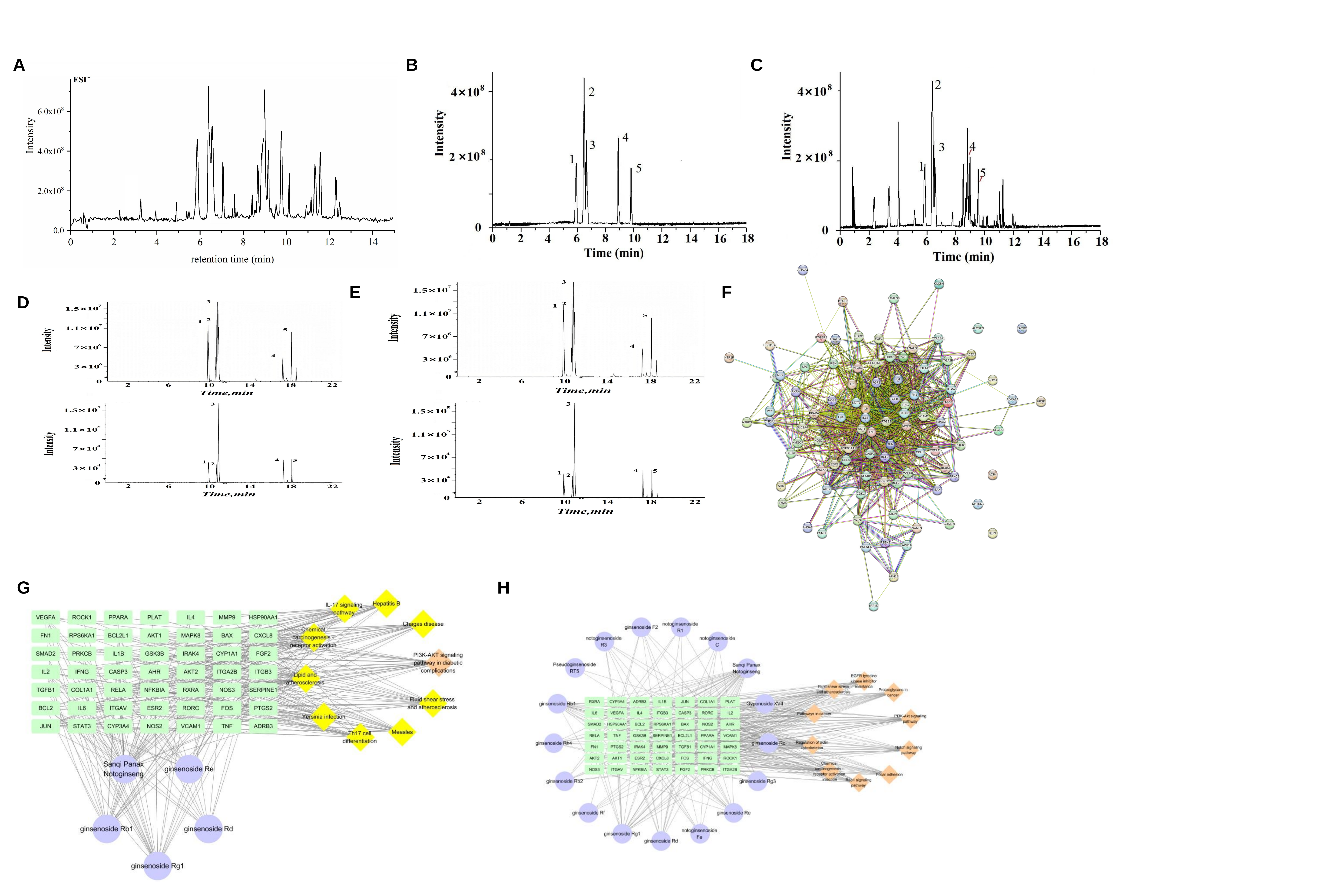

A
B
C
E
F
D
G
H

Supplement: Supplementary file 1 — Additional file 1. Supplementary Figure 1: Comprehensive analysis and pharmacological network of PNS in UVB-induced skin sunburn injury. (A) TIC of qualitative analysis for PNS . (B) TIC of reference standards for PNS (1: NGR1; 2: Ginsenoside Rg1; 3: Ginsenoside Re; 4: Ginsenoside Rb1; 5: Ginsenoside Rd). (C) Representative TIC of test samples analyzed in negative ion mode. (D, E) UHPLC-Q-Trap-MS/MS analysis of PNS components (1-5 as labeled) in negative ion mode, including fragmentation pathways and corresponding mass spectra for NGR1. (F) PPI network of shared targets between PNS and UVB-induced skin sunburn injury. (G) Key pathways and target network of PNS in treating UVB-induced skin sunburn injury. (H) Component-target network diagram illustrating interactions between 15 PNS saponins and their molecular targets in UVB-induced skin sunburn injury. Supplementary Figure 2: Functional analysis of NAT10 and saponin effects on HaCaT cell viability. (A) Immunofluorescence imaging of GFP and mCherry expression in HaCaT cells transfected with siRNA-control or siRNA-NAT10. (B) Time-dependent cell viability assay of HaCaT cells treated with 250 μM PNS at 0, 24, 48, 72, and 96 hours. (C) Cell viability of HaCaT cells following treatment with 250 μM NGR1 across the same time intervals (0–96 h). Data represents mean±S.D. from three independent experiments. Statistical significance was determined using Student’s t-test with N.S. non-significant differences, * P<0.05, ** P<0.01 indicating different levels of significance. [file 13020_2025_1270_MOESM1_ESM.pptx]

## Slide 1
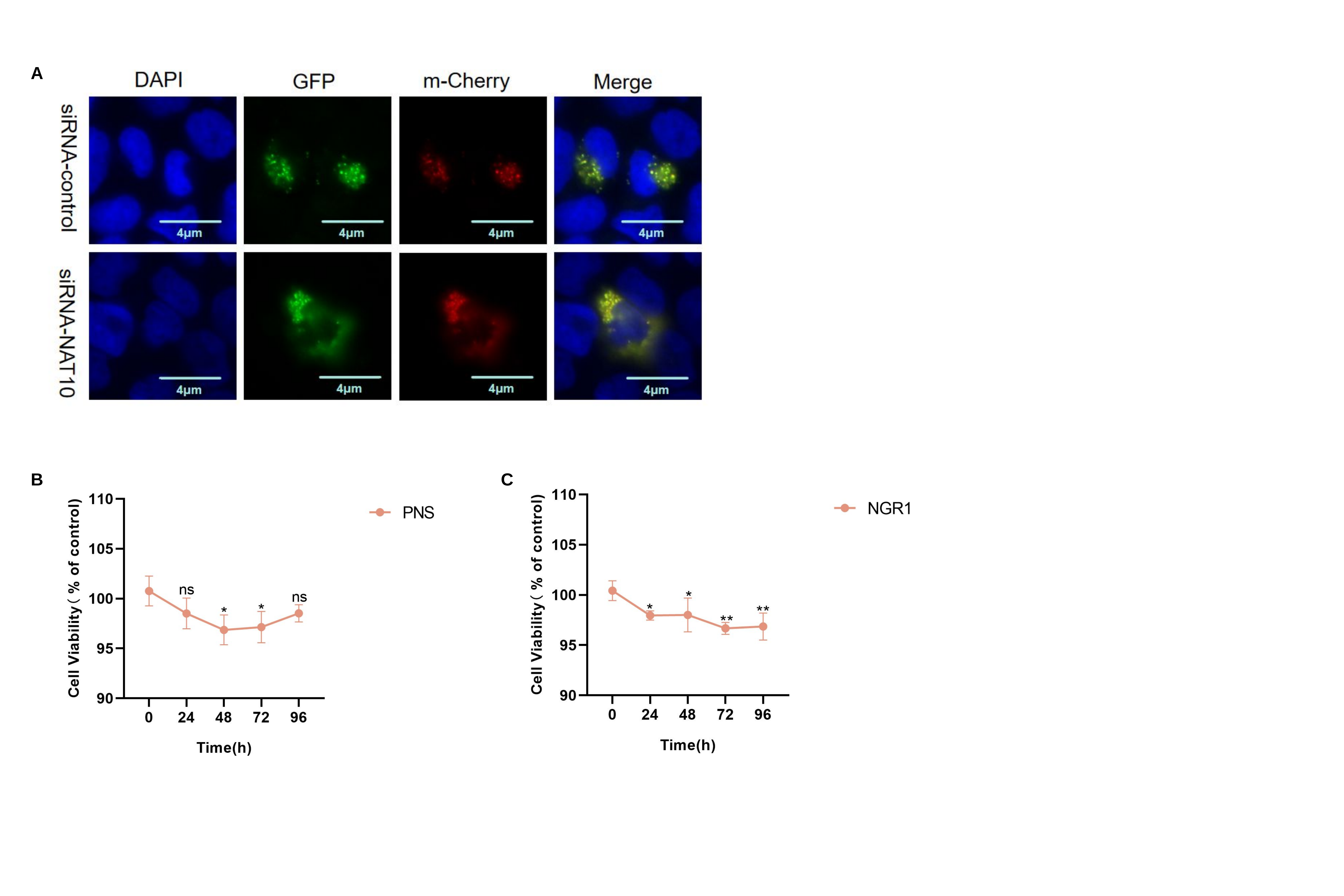

A
B
C

Supplement: Supplementary file 2 — Additional file 2. [file 13020_2025_1270_MOESM2_ESM.pptx]
